# Likelihood-free inference via classification

**DOI:** 10.1007/s11222-017-9738-6

**Published:** 2017-03-13

**Authors:** Michael U. Gutmann, Ritabrata Dutta, Samuel Kaski, Jukka Corander

**Affiliations:** 1 School of Informatics, University of Edinburgh, Edinburgh, UK; 2 InterDisciplinary Institute of Data Science, Universitá della Svizzera italiana, Lugano, Switzerland; 3 Helsinki Institute for Information Technology, Department of Computer Science, Aalto University, Espoo, Finland; 4 Department of Biostatistics, University of Oslo, Oslo, Norway; 5 Helsinki Institute for Information Technology, Department of Mathematics and Statistics, University of Helsinki, Helsinki, Finland

**Keywords:** Approximate Bayesian computation, Generative models, Intractable likelihood, Latent variable models, Simulator-based models

## Abstract

Increasingly complex generative models are being used across disciplines as they allow for realistic characterization of data, but a common difficulty with them is the prohibitively large computational cost to evaluate the likelihood function and thus to perform likelihood-based statistical inference. A likelihood-free inference framework has emerged where the parameters are identified by finding values that yield simulated data resembling the observed data. While widely applicable, a major difficulty in this framework is how to measure the discrepancy between the simulated and observed data. Transforming the original problem into a problem of classifying the data into simulated versus observed, we find that classification accuracy can be used to assess the discrepancy. The complete arsenal of classification methods becomes thereby available for inference of intractable generative models. We validate our approach using theory and simulations for both point estimation and Bayesian inference, and demonstrate its use on real data by inferring an individual-based epidemiological model for bacterial infections in child care centers.

**Electronic supplementary material:**

The online version of this article (doi:10.1007/s11222-017-9738-6) contains supplementary material, which is available to authorized users.

## Introduction

The likelihood function plays a central role in statistical inference by quantifying to which extent some values of the model parameters are consistent with the observed data. For complex models, however, evaluating the likelihood function can be computationally very costly, which often prevents its use in practice. This paper is about statistical inference for generative models whose likelihood function cannot be computed in a reasonable time.^1^

A generative model is here defined as a parametrized probabilistic mechanism which specifies how the data are generated. It is usually implemented as a computer program that takes a state of the random number generator and some values of the model parameters $\varvec{\theta }$ as input and that returns simulated data $\mathbf {Y}_{\varvec{\theta }}$ as output. The mapping from the parameters $\varvec{\theta }$ to simulated data $\mathbf {Y}_{\varvec{\theta }}$ is stochastic, and running the computer program for different states of the random number generator corresponds to sampling from the model. Generative models are also known as simulator- or simulation-based models (Hartig et al. 2011), or implicit models (Diggle and Gratton 1984), and are closely related to probabilistic programs (Mansinghka et al. 2013). Their scope of applicability is extremely wide ranging from genetics and ecology (Beaumont 2010) to economics (Gouriéroux et al. 1993), physics (Cameron and Pettitt 2012), and computer vision (Zhu et al. 2009).

A disadvantage of complex generative models is the difficulty of performing inference with them: evaluating the likelihood function involves computing the probability of the observed data $\mathbf {X}$ as function of the model parameters $\varvec{\theta }$, which for complex models cannot be done analytically or computationally within practical time limits.

As generative models are widely used, solutions have emerged in multiple fields to perform “likelihood-free” inference, that is, inference which does not rely on the availability of the likelihood function. Approximate Bayesian computation (ABC) stems from research in genetics (Beaumont et al. 2002; Marjoram et al. 2003; Pritchard et al. 1999; Tavaré et al. 1997), while the method of simulated moments (McFadden 1989; Pakes and Pollard 1989) and indirect inference (Gouriéroux et al. 1993; Smith 2008) come from econometrics. The latter methods are traditionally used in a classical inference framework while ABC has its roots in Bayesian inference, but the boundaries have started to blur (Drovandi et al. 2011). Despite their differences, the methods all share the basic idea to perform inference about $\varvec{\theta }$ by identifying values which generate simulated data $\mathbf {Y}_{\varvec{\theta }}$ that resemble the observed data $\mathbf {X}$.

The discrepancy between the simulated and observed data is typically measured by reducing each data set to a vector of summary statistics and measuring the distance between them. Both the distance function used and the summary statistics are critical for the success of the inference procedure (see, for example, the reviews by Lintusaari et al. (2017) and Marin et al. (2012). Traditionally, researchers choose the two quantities subjectively, relying on expert knowledge about the observed data. The goal of this paper is to show that the complete arsenal of classification methods can be brought to our disposal to measure the discrepancy, and thus to perform inference for intractable generative models.

The paper is based on the observation that distinguishing two data sets that were generated with very different values of $\varvec{\theta }$ is usually easier than distinguishing two data sets that were generated with similar values. We propose to use the discriminability (classifiability) of the observed and simulated data as a discrepancy measure in likelihood-free inference.

**Fig. 1:**
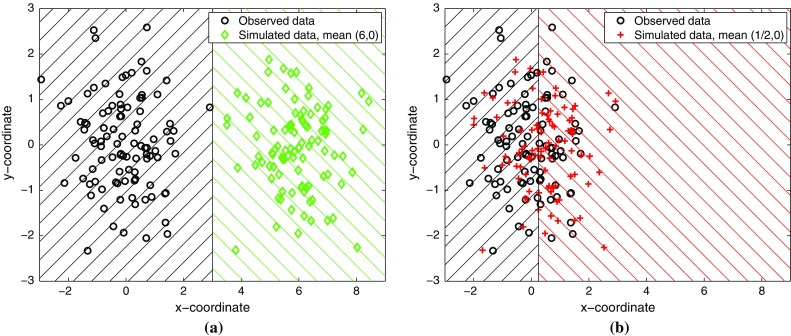
Discriminability as discrepancy measure. The observed data $\mathbf {X}$ are shown as black circles and were generated with mean $\varvec{\theta }^{\circ }= (0,0)$. The *hatched areas* indicate the Bayes classification rules. **a** High discriminability: Simulated data $\mathbf {Y}_{\varvec{\theta }}$ (*green diamonds*) were generated with $\varvec{\theta }=(6,0)$. **b** Low discriminability: $\mathbf {Y}_{\varvec{\theta }}$ (*red crosses*) were generated with $\varvec{\theta }= (1/2,0)$. As $\varvec{\theta }$ approaches $\varvec{\theta }^{\circ }$, the discriminability (best classification accuracy) of $\mathbf {X}$ and $\mathbf {Y}_{\varvec{\theta }}$ drops. We propose to use the discriminability as discrepancy measure for likelihood-free inference

We visualize the basic idea in Fig. 1 for the inference of the mean $\varvec{\theta }$ of a bivariate Gaussian with identity covariance matrix. The observed data $\mathbf {X}$, shown with black circles, were generated with mean $\varvec{\theta }^{\circ }$ equal to zero. Figure 1a shows that data $\mathbf {Y}_{\varvec{\theta }}$ simulated with mean $\varvec{\theta }=(6,0)$ can be easily distinguished from $\mathbf {X}$. The indicated classification rule yields an accuracy of 100%. In Fig. 1b, on the other hand, the data were simulated with $\varvec{\theta }= (1/2,0)$ and distinguishing such data from $\mathbf {X}$ is much more difficult; the best classification rule only yields 58% correct assignments. Moreover, if the data were simulated with $\varvec{\theta }= \varvec{\theta }^{\circ }$, the classification task could not be solved significantly above chance level. This suggests that we can perform likelihood-free inference by identifying parameters which yield chance-level discriminability only.

The remaining parts of the paper are structured as follows: In Sect. 2, we flesh out the basic idea. We then show in Sects. 3 and 4 how classification allows us to perform statistical inference of generative models in both a classical and Bayesian framework. The approach will be validated on continuous, binary, discrete, and time series data where ground truth is known. In Sect. 5, we apply the methodology to real data, and in Sect. 6, we discuss the proposed approach and related work. Section 7 concludes the paper.

## Measuring discrepancy via classification

Standard classification methods operate on feature vectors that numerically represent the properties of the data that are judged relevant for the discrimination task (Hastie et al. 2009; Wasserman 2004). There is some freedom in how the feature vectors are constructed. In the simplest case, the data are statistically independent and identically distributed (iid) random variables, and the features are equal to the data points, as in Fig. 1. But the approach of using classification to measure the discrepancy is not restricted to iid data. In the paper, we will construct features and set up a classification problems also for time series or matrix-valued data.

We denote the feature vectors from the observed data $\mathbf {X}$ by $\mathbf {x}_i$, and the feature vectors from the simulated data $\mathbf {Y}_{\varvec{\theta }}$ by $\mathbf {y}_i$, where the dependency on $\varvec{\theta }$ is suppressed for notational simplicity. We assume that we obtained *n* feature vectors from each of the two data sets. The $\mathbf {x}_i$ are then associated with class label 0 and the $\mathbf {y}_i$ with class label 1, which yields the augmented data set $\mathcal {D}_{\varvec{\theta }}$,

1 $$\begin{aligned} \mathcal {D}_{\varvec{\theta }}= \{(\mathbf {x}_1,0),\ldots ,(\mathbf {x}_n,0),(\mathbf {y}_1,1),\ldots ,(\mathbf {y}_n,1)\}. \end{aligned}$$

Classification consists in predicting the class labels of the features in $\mathcal {D}_{\varvec{\theta }}$. This is done by means of a classification rule *h* that maps each feature vector $\mathbf {u}$ to its class label $h(\mathbf {u}) \in \{0,1\}$. The performance of *h* on $\mathcal {D}_{\varvec{\theta }}$ can be assessed by the classification accuracy $\text {CA}$,

2 $$\begin{aligned} \text {CA}(h,\mathcal {D}_{\varvec{\theta }}) = \frac{1}{2n}\left( \sum _{i=1}^{n}[1-h(\mathbf {x}_i)]+h(\mathbf {y}_i)\right) , \end{aligned}$$

which is the proportion of correct assignments. The largest classification accuracy on average is achieved by the Bayes classification rule $h^{*}_{\varvec{\theta }}$, which consists in assigning a feature vector to $\mathbf {X}$ if it is more probable that the feature belongs to $\mathbf {X}$ than to $\mathbf {Y}_{\varvec{\theta }}$, and vice versa for $\mathbf {Y}_{\varvec{\theta }}$ (Hastie et al. 2009; Wasserman 2004). We denote this largest classification accuracy by $J_n^{*}(\varvec{\theta })$,

3 $$\begin{aligned} J_n^{*}(\varvec{\theta }) = \text {CA}\left( h^{*}_{\varvec{\theta }},\mathcal {D}_{\varvec{\theta }})\right. \end{aligned}$$

It is an indicator of the discriminability (classifiability) of $\mathbf {X}$ and $\mathbf {Y}_{\varvec{\theta }}$.

In the motivating example in Fig. 1, the labels of the data points are indicated by their markers, and the Bayes classification rule by the hatched areas. The classification accuracy $J_n^{*}(\varvec{\theta })$ decreases from 100% (perfect classification performance) toward 50% (chance-level performance) as $\varvec{\theta }$ approaches $\varvec{\theta }^{\circ }$, the parameter value which was used to generate the observed data $\mathbf {X}$. While this provides an intuitive justification for using $J_n^{*}(\varvec{\theta })$ as discrepancy measure, an analytical justification will be given in the next section where we show that $J_n^{*}(\varvec{\theta })$ is related to the total variation distance under mild conditions.

In practice, $J_n^{*}(\varvec{\theta })$ is not computable because the Bayes classification rule $h^{*}_{\varvec{\theta }}$ involves the probability distribution of the data which is unknown in the first place. But the classification literature provides a wealth of methods to learn an approximation $\hat{h}_{\varvec{\theta }}$ of the Bayes classification rule, and $J_n^{*}(\varvec{\theta })$ can be estimated via cross-validation (Hastie et al. 2009; Wasserman 2004).

We will use several straightforward methods to obtain $\hat{h}_{\varvec{\theta }}$: linear discriminant analysis (LDA), quadratic discriminant analysis (QDA), $L_1$-regularized polynomial logistic regression, $L_1$-regularized polynomial support vector machine (SVM) classification, and an aggregation of the above and other methods (max-rule, see Supplementary material 1.1). These are by no means the only applicable methods. In fact, any method yielding a good approximation of $h^{*}_{\varvec{\theta }}$ may be chosen; our approach makes the complete arsenal of classification methods available for inference of generative models.

While other approaches are possible, for the approximation of $J_n^{*}(\varvec{\theta })$, we use *K*-fold cross-validation where the data $\mathcal {D}_{\varvec{\theta }}$ are divided into *K* folds of training and validation sets, the different validation sets being disjoint. The training sets are used to learn the classification rules $\hat{h}_{\varvec{\theta }}^k$ by any of the methods above, and the validation sets $\mathcal {D}_{\varvec{\theta }}^k$ are used to measure their performances $\text {CA}(\hat{h}_{\varvec{\theta }}^k,\mathcal {D}_{\varvec{\theta }}^k)$. The average classification accuracy on the validation sets, $J_n(\varvec{\theta })$,

4 $$\begin{aligned} J_n(\varvec{\theta }) = \frac{1}{K}\sum _{k=1}^K \text {CA}\left( \hat{h}_{\varvec{\theta }}^k,\mathcal {D}_{\varvec{\theta }}^k\right) , \end{aligned}$$

approximates $J_n^{*}(\varvec{\theta })$ and is used as computable measure of the discrepancy between $\mathbf {X}$ and $\mathbf {Y}_{\varvec{\theta }}$.

We used $K = 5$ folds in the paper. In cross-validation, large values of *K* generally lead to approximations with smaller bias but larger variance than small values of *K*. Intermediate values like $K=5$ are thought to lead to a good balance between the two desiderata (e.g., Hastie et al. 2009, Section 7.10).

We next show on a range of different kinds of data that most of the different classification methods yield equally good approximations of $J_n^{*}(\varvec{\theta })$ for large sample sizes. Continuous data (drawn from a univariate Gaussian distribution of variance one), binary data (from a Bernoulli distribution), count data (from a Poisson distribution), and time series data (from a zero mean moving average model of order one) are considered. For the first three data sets, the unknown parameter is the mean, and for the moving average model, the lag coefficient is the unknown quantity (see Supplementary material 1.2 for the model specifications). Unlike for the other three data sets, the data points from the moving average model are not statistically independent, as the lag coefficient affects the correlation between two consecutive time points $x_t$ and $x_{t+1}$. For the classification, we treated each pair $(x_t,x_{t+1})$ as a feature.

**Fig. 2:**
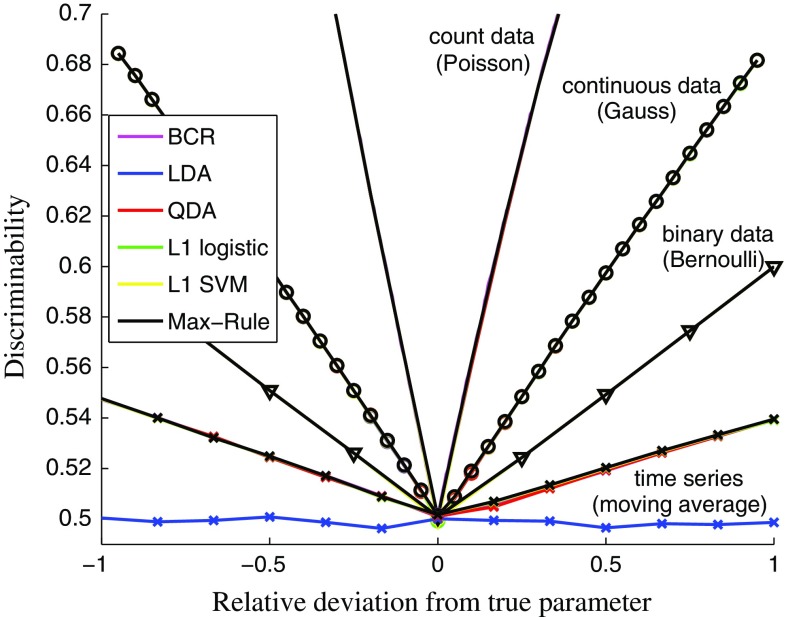
Comparison of the classification accuracy of the Bayes and the learned classification rules for large sample sizes ($n={100{,}000}$). The symmetric curves depict $J_n$ and $J_n^{*}$ as a function of the relative deviation of the model parameter from the true data-generating parameter. As the curves of the different methods are indistinguishable, quadratic discriminant analysis (QDA), $L_1$-regularized polynomial logistic regression (L1 logistic), $L_1$-regularized polynomial support vector machine classification (L1 SVM), and a max-combination of these and other methods (max-rule) perform as well as the Bayes classification rule, which assumes the true distributions to be known (BCR). For linear discriminant analysis (LDA), this holds with the exception of the moving average model

Figure 2 shows that for the Gaussian, Bernoulli, and Poisson data, all the considered classification methods perform as well as the Bayes classification rule (BCR), yielding discrepancy measures $J_n(\varvec{\theta })$ that are practically identical to $J_n^{*}(\varvec{\theta })$. The same holds for the moving average model, with the exception of LDA. The reason is that LDA is not sensitive to the correlation between $x_t$ and $x_{t+1}$, which would be needed to discover the value of the lag coefficient. In other words, the Bayes classification rule $h^{*}_{\varvec{\theta }}$ is outside the family of possible classification rules learned by LDA.

The examples show that classification can be used to identify the data-generating parameter value $\varvec{\theta }^{\circ }$ by minimizing $J_n(\varvec{\theta })$. Further evidence is provided as Supplementary material 2. The derivation of conditions which guarantee the identification of $\varvec{\theta }^{\circ }$ via classification in general is the topic of the next section.

**Fig. 3:**
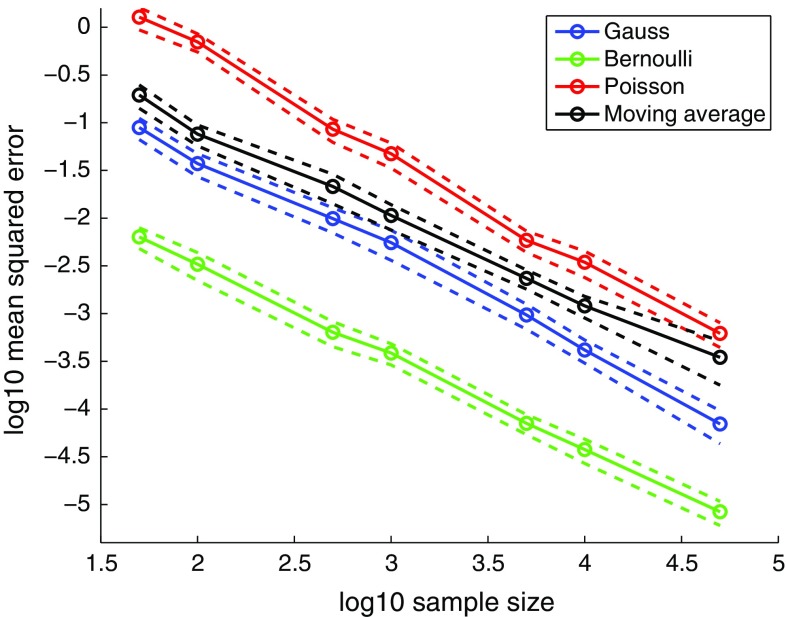
Empirical evidence for consistency. The figure shows the mean squared estimation error ${{\mathrm{E}}}[||\varvec{\hat{\theta }}_n-\varvec{\theta }^{\circ }||^2]$ for the examples in Fig. 2 as a function of the sample size *n* (*solid lines*, *circles*). The mean was computed as an average over 100 outcomes. The *dashed lines* depict the mean ±2 standard errors. The linear trend on the log–log scale suggests convergence in quadratic mean, and hence consistency of the estimator $\varvec{\hat{\theta }}_n$. The results are for $L_1$-regularized logistic regression, see Supplementary material 3 for the other classification methods

## Classical inference via classification

In this section, we consider the task of finding the single best parameter value. This can be the primary goal of the inference or only the first step before computing the posterior distribution, which will be considered in the following section. In our context, the best parameter value is the value for which the simulated data $\mathbf {Y}_{\varvec{\theta }}$ are the least distinguishable from the observed data $\mathbf {X}$, that is, the parameter $\varvec{\hat{\theta }}_n$ which minimizes $J_n$,

5 $$\begin{aligned} \varvec{\hat{\theta }}_n = \text {argmin}_{\varvec{\theta }} J_n(\varvec{\theta }). \end{aligned}$$

We show that $\varvec{\hat{\theta }}_n$ is a consistent estimator: Assuming that the observed data $\mathbf {X}$ equal some $\mathbf {Y}_{\varvec{\theta }^{\circ }}$, generated with unknown parameter $\varvec{\theta }^{\circ }$, conditions are given under which $\varvec{\hat{\theta }}_n$ converges to $\varvec{\theta }^{\circ }$ in probability as the sample size *n* increases. Figure 3 provides motivating evidence for consistency of $\varvec{\hat{\theta }}_n$.

The proposition below lists two conditions. The first one is related to convergence of frequencies to expectations (law of large numbers), the second to the ability to learn the Bayes classification rule more accurately as the sample size increases. We prove the proposition in “Appendix.” Some basic assumptions are made: The $\mathbf {x}_i$ are assumed to have the marginal probability measure ${\text {P}}_{\varvec{\theta }^{\circ }}$ and the $\mathbf {y}_i$ the marginal probability measure ${\text {P}}_{\varvec{\theta }}$ for all *i*, which amounts to a weak stationarity assumption. Importantly, the stationarity assumption does not rule out statistical dependencies between the data points; time series data, for example, are allowed. We also assume that the parametrization of ${\text {P}}_{\varvec{\theta }}$ is not degenerate, that is, there is a compact set ${\varTheta }$ containing $\varvec{\theta }^{\circ }$ where $\varvec{\theta }\ne \varvec{\theta }^{\circ }$ implies that ${\text {P}}_{\varvec{\theta }} \ne P_{\varvec{\theta }^{\circ }}$.

### Proposition 1

Denote the set of features which the Bayes classification rule $h^{*}_{\varvec{\theta }}$ classifies as being from the simulated data by $H_{\varvec{\theta }}^{*}$. The expected discriminability ${{\mathrm{E}}}(J_n^{*}(\varvec{\theta }))$ equals $J(\varvec{\theta })$,

6 $$\begin{aligned} J(\varvec{\theta }) = \frac{1}{2} + \frac{1}{2}\left( {\text {P}}_{\varvec{\theta }}\left( H^{*}_{\varvec{\theta }}\right) - {\text {P}}_{\varvec{\theta }^{\circ }}\left( H^{*}_{\varvec{\theta }}\right) \right) , \end{aligned}$$

and $\varvec{\hat{\theta }}_n$ converges to $\varvec{\theta }^{\circ }$ in probability as the sample size *n* increases, $\varvec{\hat{\theta }}_n \mathop {\rightarrow }\limits ^{P}\varvec{\theta }^{\circ }$, if

7 $$\begin{aligned}&\sup _{\varvec{\theta }\in {\varTheta }} \left| J_n^{*}(\varvec{\theta })-J(\varvec{\theta })\right| \mathop {\rightarrow }\limits ^{P}0 \;\; \text {and} \end{aligned}$$

8 $$\begin{aligned}&\sup _{\varvec{\theta }\in {\varTheta }} \left| J_n(\varvec{\theta })-J_n^{*}(\varvec{\theta })\right| \mathop {\rightarrow }\limits ^{P}0. \end{aligned}$$

The two conditions guarantee that $J_n(\varvec{\theta })$ converges uniformly to $J(\varvec{\theta })$, so that $J(\varvec{\theta })$ is minimized with the minimization of $J_n(\varvec{\theta })$ as *n* increases. Since $J(\varvec{\theta })$ attains its minimum at $\varvec{\theta }^{\circ }$, $\varvec{\hat{\theta }}_n$ converges to $\varvec{\theta }^{\circ }$. By definition of $H^{*}_{\varvec{\theta }}$, ${\text {P}}_{\varvec{\theta }}(H^{*}_{\varvec{\theta }}) - {\text {P}}_{\varvec{\theta }^{\circ }}(H^{*}_{\varvec{\theta }})$ is one half of the total variation distance between the two distributions (Pollard 2001, Chapter 3). The limiting objective $J(\varvec{\theta })$ corresponds thus to a well-defined statistical distance between ${\text {P}}_{\varvec{\theta }}$ and ${\text {P}}_{\varvec{\theta }^{\circ }}$.

The condition in Eq. (7) is about convergence of sample averages to expectations. Standard convergence results apply for statistically independent features. For features with statistical dependencies, e.g., time series data, corresponding convergence results are investigated in empirical process theory (van der Vaart and Wellner 1996), which forms a natural limit of what is studied in this paper. We may only note that by definition of *J*, convergence will depend on the complexity of the sets $H^{*}_{\varvec{\theta }}$, $\varvec{\theta }\in {\varTheta }$, and hence the complexity of the Bayes classification rules $h^{*}_{\varvec{\theta }}$. The condition does not depend on the classification method employed. In other words, this first condition is about the difficulty of the classification problems that need to be solved. The condition in Eq. (8), on the other hand, is about the ability to solve them: The performance of the learned rule needs to approach the performance of the Bayes classification rule as the number of available samples increases. How to best learn such rules and finding conditions which guarantee successful learning is a research area in itself (Zhang 2004).

In Fig. 2, LDA did not satisfy the condition in Eq. (8) for the moving average data, which can be seen by the chance-level performance for all parameters tested. This failure of LDA suggests a practical means to test whether the second condition holds: We generate data sets with two very different parameter values so that it is unlikely that the data sets are similar to each other, and learn to discriminate between them. If the performance is persistently close to chance level, the Bayes classification rule is likely outside the family of classification rules that the method is able to learn, so that the condition would be violated. Regarding the first condition, the results in Fig. 3 suggest that it is satisfied for all four inference problems considered. Generally verifying whether the sample average converges to the expectation, e.g., via a general method that works reliably for any kind of time series data, seems, however, difficult.

**Fig. 4:**
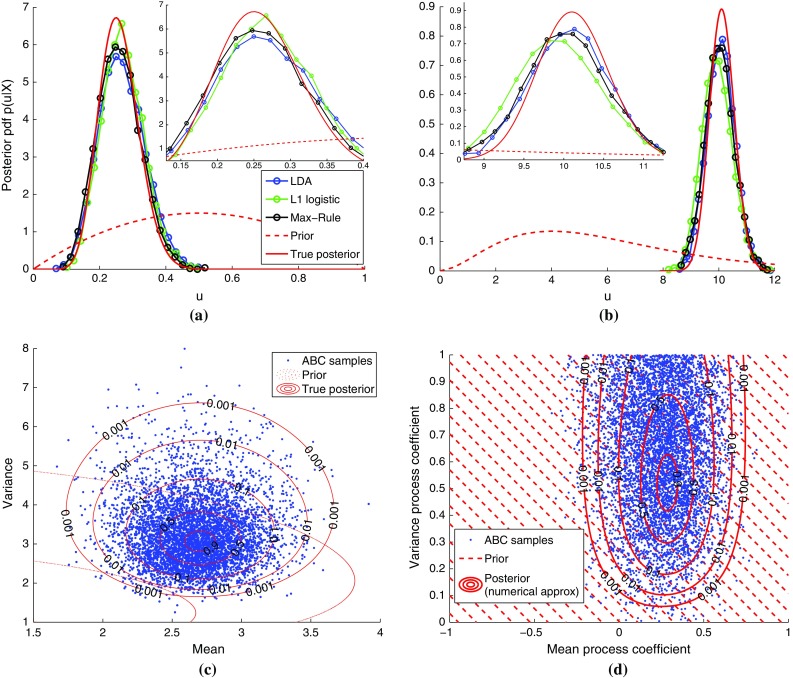
Posterior distributions inferred by classifier ABC for binary, count, continuous, and time series data. The results are for 10,000 ABC samples and $n=50$. For the univariate cases, the samples are summarized as empirical pdfs. For the bivariate cases, scatter plots of the obtained samples are shown (the results are for the max-rule). The numbers on the contours are relative to the maximum of the reference posterior. For the autoregressive conditional heteroskedasticity (ARCH) model, the hatched area indicates the domain of the uniform prior. Supplementary material 4 contains additional examples and results. **a** Binary data (Bernoulli), **b** count data (Poisson), **c** continuous data (Gauss), and **d** time series (ARCH)

## Bayesian inference via classification

We consider next inference of the posterior distribution of $\varvec{\theta }$ in the framework of approximate Bayesian computation (ABC).

ABC comprises several simulation-based methods to obtain samples from the posterior distribution when the likelihood function is not known (for review papers, see, e.g., Lintusaari et al. 2017; Marin et al. 2012). ABC algorithms are iterative: The basic steps at each iteration are as follows:

1. Proposing a parameter value $\varvec{\theta }'$,
2. Simulating pseudo-observed data $\mathbf {Y}_{\varvec{\theta }'}$, and then
3. Accepting or rejecting the proposal based on a comparison of $\mathbf {Y}_{\varvec{\theta }'}$ with the real observed data $\mathbf {X}$.

How to actually measure the discrepancy between the observed and the simulated data is a major difficulty in these methods (Lintusaari et al. 2017; Marin et al. 2012). We here show that $J_n$ can be used as a discrepancy measure in ABC; in the following, we call this approach “classifier ABC.” In step 3, we thus compare $\mathbf {Y}_{\varvec{\theta }'}$ and $\mathbf {X}$ through the lenses of a classifier by computing the discriminability of the two data sets.

The results reported in this paper were obtained with a sequential Monte Carlo implementation (see Supplementary material 1.3). The use of $J_n$ in ABC is, however, not restricted to that particular algorithm.

We validated classifier ABC on binary (Bernoulli), count (Poisson), continuous (Gaussian), and time series (ARCH) data (see Supplementary material 1.2 for the model details). The true posterior for the autoregressive conditional heteroskedasticity (ARCH) model is not available in closed form. We approximated it using deterministic numerical integration, as detailed in Supplementary material 1.2.

The inferred empirical posterior probability density functions (pdfs) are shown in Fig. 4. There is a good match with the true posterior pdfs or the approximation obtained with deterministic numerical integration. Different classification methods yield different results, but the overall performance is rather similar. Regarding computation time, the simpler LDA and QDA tend to be faster than the other classification methods used, with the max-rule being the slowest one. Additional examples as well as links to movies showing the evolution of the posterior samples in the ABC algorithm can be found in Supplementary material 4.

As a quantitative analysis, we computed the relative error of the posterior means and standard deviations. The results, reported as part of Supplementary material 4, show that the errors in the posterior mean are within 5% after five iterations of the ABC algorithm for the examples with independent data points. For the time series, where the data points are not independent, a larger error of 15% occurs. The histograms and scatter plots show, however, that the corresponding ABC samples are still very reasonable.

## Application on real data

We next used our approach to infer an intractable model of bacterial infections in child care centers.

### Data and model

The observed data $\mathbf {X}$ were the presence or absence of different strains of the bacterium *Streptococcus pneumoniae* among attendees of $M=29$ child care centers in the metropolitan area of Oslo, Norway, at single points of time $T_m$ (cross-sectional data). On average, $N = 53$ children attended a center. Only a subset of size $N_m$ of all attendees of each center was sampled. The data were collected and first described by Vestrheim et al. (2008).

In the following, we represent the colonization state of individual *i* in a child care center by the binary variable $I_{is}^{t}, s=1,\ldots ,S$, where *S* the total number of strains in circulation. If the attendee is infected with strain *s* of the bacterium at time *t*, $I_{is}^t=1$, and otherwise, $I_{is}^t=0$. The observed data $\mathbf {X}$ consisted thus of a set of $M=29$ binary matrices of size $N_m \times S$ formed by the $I_{is}^{T_m}$, $i=1,\ldots ,N_m, s=1,\ldots ,S$.

**Fig. 5:**
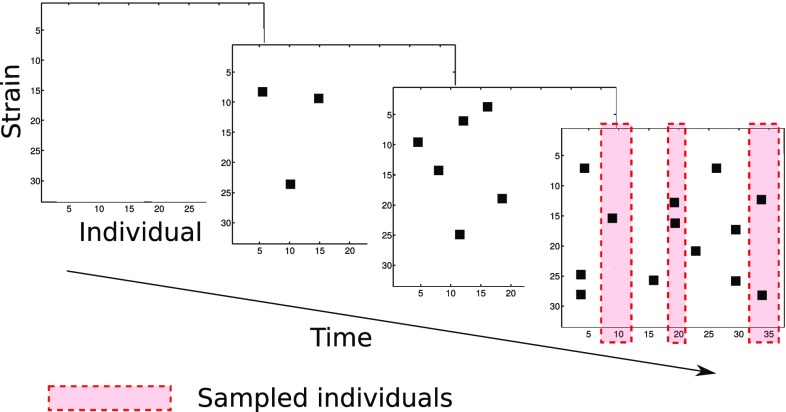
Sketch of the individual-based epidemic model. The evolution of the colonization states in a single child care center is shown. Colonization is indicated by the *black squares*

The model for which we performed inference was developed by Numminen et al. (2013). It is individual-based and consists of a continuous-time Markov chain for the transmission dynamics inside a child care center paired with an observation model. The child care centers were assumed independent. The model is sketched in Fig. 5 for a single center.

In each child care center, the transmission dynamics started with zero infected individuals, $I_{is}^{0}=0$ for all *i* and *s*, after which the states evolved in a stochastic manner according to the following transition probabilities:

9 $$\begin{aligned}&{\text {P}}\left( I_{is}^{t+h}=0 | I_{is}^t =1\right) = h +o(h), \end{aligned}$$

10 $$\begin{aligned}&{\text {P}}\left( I_{is}^{t+h}=1 | I_{is'}^t =0 \; \forall s'\right) = R_s^t h +o(h), \end{aligned}$$

11 $$\begin{aligned}&{\text {P}}\left( I_{is}^{t+h}\!=\!1 | I_{is}^t \!=\!0, \; \exists s': I_{is'}^t=1\right) \!=\! \theta R_s^t h+o(h), \end{aligned}$$

where *h* is a small time interval and *o*(*h*) a remainder term satisfying $\lim _{h\rightarrow 0} o(h)/h = 0$. Equation (9) describes the probability to clear strain *s*, Eq. (10) the probability to be infected by it when previously not infected with any strain, and Eq. (11) the probability to be infected by it when previously infected with another strain $s'$. The rate of infection with strain *s* at time *t* is denoted by $R_s^t$, and $\theta \in (0,1)$ is an unknown co-infection parameter. For $\theta = 0$, the probability for a co-infection is zero. The rate $R_s^t$ was modeled as

12 $$\begin{aligned} R_s^t&= \beta E_s^t +{\varLambda }P_s, \end{aligned}$$

13 $$\begin{aligned} E_s^t&= \sum _{j=1}^N \frac{1}{N-1} \frac{I_{js}^t}{n_j^t}, \end{aligned}$$

14 $$\begin{aligned} n_j^t&= \sum _{s'=1}^S I_{js'}^t, \end{aligned}$$

where *N* is the average number of children attending the child care center, and ${\varLambda }$ and $\beta$ are two unknown rate parameters that scale the static probability $P_s$ for an infection happening outside the child care center and the dynamic probability $E_s^t$ for an infection from within, respectively. The probability $P_s$ and the number of strains *S* were determined by an analysis of the overall distribution of the strains in the cross-sectional data (yielding $S=33$; for $P_s$, see Numminen et al. 2013). The expression for $E_s^t$ in Eq. (13) was derived by assuming that contacts happen uniformly at random [the probability for a contact is $1/(N-1)$], and that the strains attendee *j* is carrying are all transmitted with equal probability (with $n_j^t$ being the total number of strains carried by attendee *j*, the probability for a transmission of strain *s* is $I_{js}^t/n_j^t$).

The observation model was random sampling of $N_m$ individuals without replacement from the average number *N* of individuals attending a child care center. A stationarity assumption was made so that the exact value of the sampling time $T_m$ was not of importance as long as it is sufficiently large so that the system is in its stationary regime.

The model has three parameters for which uniform priors were assumed: Parameter $\beta \in (0,11)$ which is related to the probability to be infected by someone inside a child care center, parameter ${\varLambda }\in (0,2)$ for the probability of an infection from an outside source, and parameter $\theta \in (0,1)$ which is related to the probability to be infected with multiple strains. With a slight abuse of notation, we will use $\varvec{\theta }=(\beta ,{\varLambda },\theta )$ to denote the compound parameter vector.

### Reference inference method

Since the likelihood function is intractable, the model was inferred with ABC in previous work (Numminen et al. 2013). The summary statistics were chosen based on epidemiological considerations and the distance function was adapted to the specific problem at hand.

To compare $\mathbf {X}$ and $\mathbf {Y}_{\varvec{\theta }}$, Numminen et al. (2013) first summarized each of the $M=29$ child care centers of the simulated and observed data using four statistics:

1. The strain diversity in the child care centers,
2. The number of different strains circulating,
3. The proportion of individuals who are infected, and
4. The proportion of individuals who are infected with more than one strain.

For each of the four summary statistics, the empirical cumulative distribution function (cdf) was computed from the obtained $M=29$ values. The $L_1$ distances between the empirical cdfs of the summary statistics for $\mathbf {X}$ and $\mathbf {Y}_{\varvec{\theta }}$ were then used to assess the discrepancy (Numminen et al. 2013). Inference was performed with a sequential Monte Carlo ABC algorithm with four generations. The corresponding posterior distribution will serve as reference against which we compare the solution by classifier ABC.

### Formulation as classification problem

For likelihood-free inference via standard classification, the observed matrix-valued data were transformed to feature vectors. We used simple features which reflect the matrix structure and the binary nature of the data.

For the matrix nature of the data, the rank of each matrix and the $L_2$-norm of the singular values (scaled by the size of the matrix) were used. For the binary nature of the data, we counted the fraction of ones in certain subsets of each matrix and used the average of the counts and their variability as features. The set of rows and the set of columns were used, as well as 100 randomly chosen subsets. Each random subset contained 10% of the elements of a matrix. Since the average of the counts is the same for the row and column subsets (it equals the fraction of all ones in a matrix), only one average was used.

The features $\mathbf {x}_i$ or $\mathbf {y}_i$ in the classification had thus size seven (2 dimensions are for the matrix properties, 3 dimensions for the column and row subsets, and 2 dimensions for the random subsets). Multiple random subsets can be extracted from each matrix. We made use of this to obtain $n={1000}$ features $\mathbf {x}_i$ and $\mathbf {y}_i$. We also ran classifier ABC without random subsets; the classification problems consisted then in discriminating between two data sets consisting each of 29 five-dimensional feature vectors. As classification method, we used LDA.

**Fig. 6:**
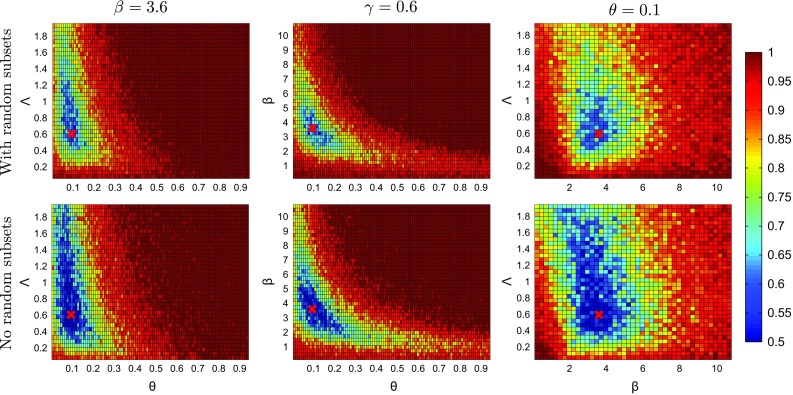
Testing the applicability of the discrepancy measure $J_n$ to infer the individual-based epidemic model. The figures show $J_n(\varvec{\theta })$ when one parameter is fixed at a time. The *red crosses* mark the data-generating parameter value $\varvec{\theta }^{\circ }= (\beta ^o,{\varLambda }^o,\theta ^o)=(3.6, 0.6, 0.1)$. The presence of random features produced more localized regions with small $J_n$

### Inference results

In ABC, the applicability of a discrepancy measure can be assessed by first performing inference on synthetic data of the same size and structure as the observed data but simulated from the model with known parameter values. Since ABC algorithms are rather time-consuming, we first tested the applicability of $J_n$ in the framework of point estimation. We computed $J_n(\varvec{\theta })$ varying only two of the three parameters at a time, keeping the third parameter fixed at the value which was used to generate the data. To eliminate random effects, we used for all $\varvec{\theta }$ the same random number generator seed when simulating the $\mathbf {Y}_{\varvec{\theta }}$. The seeds for $\mathbf {X}$ and the $\mathbf {Y}_{\varvec{\theta }}$ were different.

Figure 6 shows the results for classification with randomly chosen subsets (top row) and without (bottom row). The diagrams on the top and bottom row are very similar, both have well-defined regions in the parameter space for which $J_n$ is close to one half, which corresponds to chance-level discriminability. But the features from the random subsets were helpful to discriminate between $\mathbf {X}$ and $\mathbf {Y}_{\varvec{\theta }}$ and produced more localized regions with small $J_n$. The results suggest that LDA, the arguably simplest classification method, is suitable to infer the epidemic model.

We next applied classifier ABC on the synthetic data, using a sequential Monte Carlo ABC algorithm with four generations as previously done by Numminen et al. (2013).

**Fig. 7:**
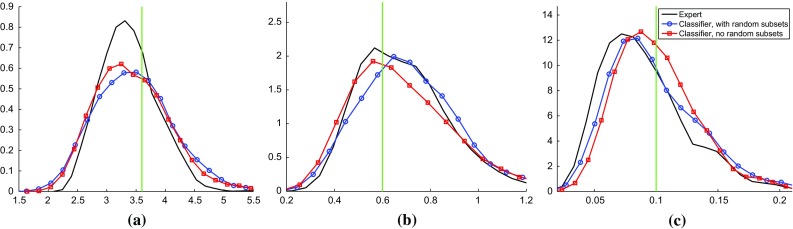
Inferring the individual-based epidemic model with classifier ABC. The results are for simulated data with known data-generating parameter $\varvec{\theta }^{\circ }$ (indicated by the *green vertical lines*). Classifier ABC with random subsets (*blue*, *circles*) or without (*red*, *squares*) both yielded posterior pdfs which are qualitatively similar to the expert solution (*black*). **a** Posterior pdf for $\beta$, **b** posterior pdf for ${\varLambda }$ and **c** posterior pdf for $\theta$

The resulting posterior pdfs are shown in Fig. 7 in the form of kernel density estimates (smoothed and scaled histograms) based on 1000 ABC samples. It can be seen that classifier ABC with or without random subsets both yielded results which are qualitatively similar to the expert solution. The strongest difference is that the tails of the posterior pdf of $\beta$ are heavier for classifier ABC than for the expert solution. In case of classifier ABC with random subsets, this difference became less pronounced when the algorithm was run for an additional fifth iteration (Supplementary material 5). For classifier ABC without random subsets, on the other hand, the difference persisted. This behavior is in line with Fig. 6 where the random features led to tighter $J_n$-diagrams. Overall, the results on synthetic data confirm the applicability of classifier ABC to infer the epidemic model.

**Fig. 8:**
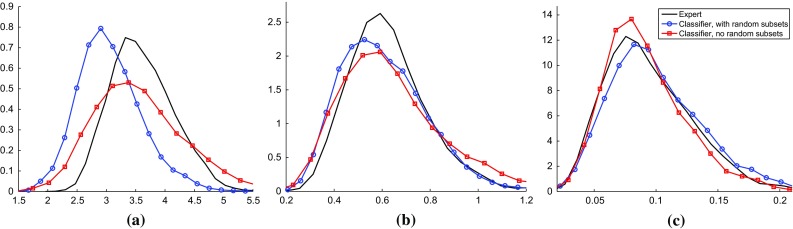
Inference results on real data, visualized as in Fig. 7. **a** Posterior pdf for $\beta$, **b** posterior pdf for ${\varLambda }$ and **c** posterior pdf for $\theta$

**Fig. 9:**
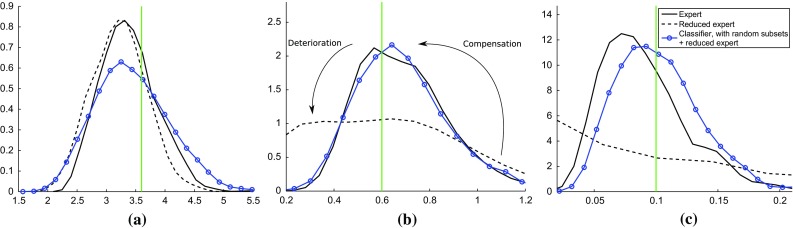
Using classifier ABC to compensate for insufficient expert statistics. The setup and visualization is as in Fig. 7. Its expert solution is reproduced for reference. Working with a reduced set of expert statistics affects the posteriors of ${\varLambda }$ and $\theta$ adversely, but classifier ABC is able to compensate (*blue curves with circles* vs. *black dashed curves*). **a** Internal infection parameter $\beta$, **b** external infection parameter ${\varLambda }$ and **c** co-infection parameter $\theta$

The results on real data are shown in Fig. 8. It can be seen that the posterior distributions obtained with classifier ABC are generally similar to the expert solution. The posterior mode of $\beta$ for classifier ABC with random subsets is slightly smaller than for the other methods. The shift could be due to stochastic variation because we only worked with 1000 ABC samples. It could, however, also be that the random features picked up some properties of the real data which the other methods are not sensitive to.

The computation time of classifier ABC with LDA was about the same as for the method by Numminen et al. (2013): On average, the total time for the data generation and the discrepancy measurement was 28.49 ± 3.45 s for LDA while it was 28.41 ± 3.45 s for the expert method; with 28.4 ± 3.45 s, most of the time was spent on generating data from the epidemic model. Altogether, classifier ABC thus yielded inference results which are equivalent to the expert solution, from both a statistical and computational point of view.

### Compensating for missing expert statistics

So far we did not use expert knowledge about the inference problem when solving it with classifier ABC. Using discriminability in a classification task as a discrepancy measure is a data-driven approach to assess the similarity between simulated and observed data. But it is not necessarily a black-box approach. Knowledge about the problem at hand can be incorporated when specifying the classification problem. Furthermore, the approach is compatible with summary statistics derived from expert knowledge: Classifier ABC, and more generally the discrepancy measure $J_n$, is able to incorporate the expert statistics by letting them be features (covariates) in the classification. The combined use of expert statistics and classifier ABC enables one to filter out properties of the model which are either not of interest or known to be wrong. Moreover, it makes the inference more robust, for example to possible misspecifications or insufficiencies of the summary statistics, as we illustrate next.

We selected two simple expert statistics used by Numminen et al. (2013), namely the number of different strains circulating and the proportion of infected individuals, and inferred the posteriors with this reduced set of summary statistics, using the method by Numminen et al. (2013) as before. Figure 9 shows that consequently, the posterior distributions of ${\varLambda }$ and $\theta$ deteriorated. The used expert statistics alone were insufficient to perform ABC. Combining the insufficient set of summary statistics with classifier ABC, however, led to a recovery of the posteriors. The result are for classifier ABC with random subsets, but the same holds for classifier ABC without random subsets (Supplementary material 5).

## Discussion

Generative models are useful and widely applicable for dealing with uncertainty and for making inferences from data. The intractability of the likelihood function is, however, often a serious problem in the inference for realistic models. While likelihood-free methods provide a powerful framework for performing inference, a limiting difficulty is the required discrepancy measurement between simulated and observed data.

We found that classification can be used to measure the discrepancy. This finding has practical value because it reduces the difficult problem of choosing an appropriate discrepancy measure to a more standard problem where we can leverage a wealth of existing solutions; whenever we can classify, we can do likelihood-free inference. It offers also theoretical value because it reveals that classification can yield consistent likelihood-free inference, and that the two fields of research, which appear very much separated at first glance, are actually tightly connected.

### Summary statistics versus features

In the proposed approach, instead of choosing summary statistics and a distance function between them as in the standard approach, we need to choose a classification method and the features. The reader may thus wonder whether we replaced one possibly arbitrary choice with another. The important point is that by choosing a classification method, we only decide about a function space, and not the classification rule itself. The classification rule that is finally used to measure the discrepancy is learned from data and is not specified by the user, which is in stark contrast to the traditional approach based on fixed summary statistics. Moreover, the function space can be chosen using cross-validation, as implemented with our max-rule, which reduces the arbitrariness even more. In Fig. 2, for example, the max-rule successfully chose to use other classification methods than LDA for the inference of the moving average model. The influence of the choice of features is also rather mild, because they only affect the discrepancy measurement via the learned classification rule. This property of the proposed approach allowed us to even use random features in the inference of the epidemic model.

The possibility to use random features, however, does not mean that we should not use reliable expert knowledge when available. Indeed, summary statistics derived from expert knowledge can be included by letting them be features (covariates) in the classification.

### Related work

In previous work, regression with the parameters $\varvec{\theta }$ as response variables was used to generate summary statistics from a larger pool of candidates (Aeschbacher et al. 2012; Fearnhead and Prangle 2012; Wegmann et al. 2009). The shared characteristic of these works and our approach is the learning of transformations of the summary statistics and the features, respectively. The criteria which drive the learning are, however, rather different.

Since the candidate statistics are a function of the simulated data $\mathbf {Y}_{\varvec{\theta }}$, we may consider the regression to provide an approximate inversion of the data generation process $\varvec{\theta }\mapsto \mathbf {Y}_{\varvec{\theta }}$. In this interpretation, the (Euclidean) distance of the summary statistics is an approximation of the (Euclidean) distance of the parameters. The optimal inversion of the data-generating process in a mean squared error sense is the conditional expectation ${{\mathrm{E}}}(\varvec{\theta }| \mathbf {Y}_{\varvec{\theta }})$. Fearnhead and Prangle (2012) showed that this conditional expectation is also the optimal summary statistic for $\mathbf {Y}_{\varvec{\theta }}$ if the goal is to infer $\varvec{\theta }^{\circ }$ as accurately as possible under a quadratic loss. Transformations based on regression are thus strongly linked to the computation of the distance between the parameters. The reason we learn transformations, on the other hand, is that we would like to approximate $J_n^{*}(\varvec{\theta })$ well, which is linked to the computation of the total variation distance between the distributions indexed by the parameters.

Classification was recently used in other work on ABC, but in a different manner. Intractable density ratios in Markov chain Monte Carlo algorithms were estimated using tools from classification (Pham et al. 2014), in particular random forests, and Pudlo et al. (2016) used random forests for model selection by learning to predict the model class from the simulated data instead of computing their posterior probabilities. This is different from using classification to define a discrepancy measure between simulated and observed data, as done here.

A particular classification method, (nonlinear) logistic regression, was used for the estimation of unnormalized models (Gutmann and Hyvärinen 2012), which are models where the probability density functions are known up to the normalizing partition function only (see Gutmann and Hyvärinen (2013a) for a review paper, and Barthelmé and Chopin (2015), Gutmann et al. (2011) and Pihlaja et al. (2010) for generalizations). Likelihood-based inference is intractable for unnormalized models, but unlike in the generative models considered here, the shape of the model-pdf is known which can be exploited in the inference.

At about the same time, we first presented our work (Gutmann et al. 2014a, b), Goodfellow et al. (2014) proposed to use nonlinear logistic regression to train a neural network such that it transforms “noise” samples into samples approximately following the same distribution as some given data set. The main difference to our work is that the method of Goodfellow et al. (2014) is a method for producing random samples while ours is a method for statistical inference.

### Sequential inference and prediction

We did not make any specific assumptions about the model or the structure of the observed data $\mathbf {X}$. An interesting special case occurs when $\mathbf {X}$ are an element $\mathbf {X}^{(t_0)}$ of a sequence of data sets $\mathbf {X}^{(t)}$ which are observed one after the other, and the generative model is specified accordingly to generate a sequence of simulated data sets.

For inference at $t_0$, we can distinguish between simulated data which were generated either before or after $\mathbf {X}^{(t_0)}$ are observed: In the former case, the simulated data are predictions about $\mathbf {X}^{(t_0)}$, and after observation of $\mathbf {X}^{(t_0)}$, likelihood-free inference about $\varvec{\theta }$ corresponds to assessing the accuracy of the predictions. That is, the discrepancy measurement converts the predictions of $\mathbf {X}^{(t_0)}$ into inferences of the causes of $\mathbf {X}^{(t_0)}$. In the latter case, each simulated data set can immediately be compared to $\mathbf {X}^{(t_0)}$ which enables efficient iterative identification of parameter values with low discrepancy (Gutmann and Corander 2016). That is, the possible causes of $\mathbf {X}^{(t_0)}$ can be explained more accurately with the benefit of hindsight.

### Relation to perception and artificial intelligence

Probabilistic modeling and inference play key roles in image understanding (Gutmann and Hyvärinen 2013b), robotics (Thrun et al. 2006), and artificial intelligence (Ghahramani 2015). Perception has been modeled as (Bayesian) inference based on a “mental” generative model of the world (e.g., Vincent 2015). In most of the literature, variational approximate inference has been used for intractable generative models, giving rise to the Helmholtz machine (Dayan et al. 1995) and to the free-energy in neuroscience (Friston 2010). But other approximate inference methods can be considered as well.

The discussion about sequential inference and prediction points to similarities between perception and likelihood-free inference or approximate Bayesian computation. It is intuitively sensible that perception would involve prediction of new sensory input given the past, as well as an assessment of the predictions and a refinement of their explanations after arrival of the data. The quality of the inference depends on the quality of the generative model and the quality of the discrepancy assessment. That is, the inference results may only be useful if the generative model of the world is rich enough to produce data resembling the observed data, and if the discrepancy measure can reliably distinguish between the “mentally” generated and the actually observed data.

We proposed to measure the discrepancy via classification, being agnostic about the particular classifier used. It is an open question how to generally best measure the classification accuracy when the data are arriving sequentially. Classifiers are, however, rather naturally part of perceptual systems. Rapid object recognition, for instance, can be achieved via feedforward multilayer classifiers (Serre et al. 2007), and there are several techniques to learn representations which facilitate classification (Bengio et al. 2013). It is thus conceivable that a given classification machinery is used for several purposes, for example to quickly recognize certain objects but also to assess the discrepancy between simulated and observed data.

## Conclusions and future work

In the paper, we proposed to measure the discrepancy in likelihood-free inference via classification. We focused on the principle and not on a particular classification method. Some methods may be particularly suited for certain models, where it may be possible to measure the discrepancy via the loss function that is used to learn the classification rule instead of the classification accuracy.

When working with the classification accuracy, we only use a single bit of information per data point. While this is little information, we showed that the approach yielded accurate posterior inferences and that it defines a consistent estimator. The Bayesian inference results were empirical, and it is likely that a more rigorous theoretical analysis will reveal that the single bit of information puts a limit on the possible closeness to the true posterior. While our empirical results suggest that other error sources may be more dominant in practice, the bottleneck can be avoided by using the current setup to identify the relevant summary statistics, or some transformation of them, and by computing the discrepancy by their Euclidean distance as in classical ABC. While this is a possible approach, in recent work, we chose another path by training the classifier on two simulated data sets whose size can be made as large as computationally possible (Dutta et al. 2016).

We here worked with a single simulated data set per parameter value. If multiple simulated data sets are available, they may be used to define an approximate likelihood function by, for example, averaging their corresponding discrepancies (see, e.g., Gutmann and Corander 2016, Section 3.3). The approximate likelihood function can then be maximized with respect to the parameters or used in place of the actual likelihood function in standard methods for posterior sampling.

Further exploration of the connection between classification and likelihood-free inference is likely to lead to practical improvements in general: Each parameter $\varvec{\theta }$, for instance, induces a classification problem. We here treated the classification problems separately, but they are actually related. First, the observed data $\mathbf {X}$ occur in all the classification problems. Second, the simulated data sets $\mathbf {Y}_{\varvec{\theta }}$ are likely to share some properties if the parameters are not too different. Taking advantage of the relation between the different classification problems may lead to both computational and statistical gains. In the classification literature, leveraging the solution of one problem to solve another one is generally known as transfer learning (Pan and Yang 2010). In the same spirit, leveraging transfer learning, or other methods from classification, seems promising to further advance likelihood-free inference.

### Electronic supplementary material

Below is the link to the electronic supplementary material.

Supplementary material 1 (pdf 3937 KB)

## Appendix: Proof of proposition 1

Proposition 1 is proved using an approach based on uniform convergence in probability of $J_n$ to a function *J* whose minimizer is $\varvec{\theta }^{\circ }$ (van der Vaart 1998). The proof has three steps: First, we identify *J*. Second, we find conditions under which *J* is minimized by $\varvec{\theta }^{\circ }$. Third, we derive conditions which imply that $J_n$ converges to *J*.

### Definition of *J*

For validation sets $\mathcal {D}_{\varvec{\theta }}^k$ consisting of 2*m* labeled features $(\mathbf {x}_i^k,0)$ and $(\mathbf {y}_i^k,1)$, $i=1,\ldots ,m$, we have by definition of $\text {CA}(h,\mathcal {D}_{\varvec{\theta }})$ in Eq. (2)

15 $$\begin{aligned} \text {CA}\left( \hat{h}_{\varvec{\theta }}^k,\mathcal {D}_{\varvec{\theta }}^k\right)&= \frac{1}{2m}\left( \sum _{i=1}^{m}\left[ 1-\hat{h}_{\varvec{\theta }}^k\left( \mathbf {x}_i^k\right) \right] +\hat{h}_{\varvec{\theta }}^k\left( \mathbf {y}_i^k\right) \right) \end{aligned}$$

16 $$\begin{aligned}&= \frac{1}{2} + \frac{1}{2m} \sum _{i=1}^m \hat{h}_{\varvec{\theta }}^k\left( \mathbf {y}_i^k\right) - \hat{h}_{\varvec{\theta }}^k\left( \mathbf {x}_i^k\right) , \end{aligned}$$

so that $J_n(\varvec{\theta })$ in Eq. (4) can be written as

17 $$\begin{aligned} J_n(\varvec{\theta })&= \frac{1}{K}\sum _{k=1}^K \left( \frac{1}{2} +\frac{1}{2m} \sum _{i=1}^m \hat{h}_{\varvec{\theta }}^k\left( \mathbf {y}_i^k\right) -\hat{h}_{\varvec{\theta }}^k\left( \mathbf {x}_i^k\right) \right) \end{aligned}$$

18 $$\begin{aligned}&=\frac{1}{2} + \frac{1}{2Km} \sum _{i=1}^m \sum _{k=1}^K \hat{h}_{\varvec{\theta }}^k\left( \mathbf {y}_i^k\right) - \hat{h}_{\varvec{\theta }}^k\left( \mathbf {x}_i^k\right) . \end{aligned}$$

Each feature is used exactly once for validation since the $\mathcal {D}_{\varvec{\theta }}^k$ are disjoint. We make the simplifying assumption that splitting the original *n* features into *K* folds of *m* features was possible without remainders. We can then order the $\mathbf {y}_i^k$ as

$$\begin{aligned} \mathbf {y}_1^1,\ldots ,\mathbf {y}_m^1,\mathbf {y}_1^2,\ldots ,\mathbf {y}_m^2,\mathbf {y}_1^3,\ldots ,\mathbf {y}_m^K, \end{aligned}$$

and relabel them from 1 to *n*. Doing the same for the $\mathbf {x}_i^k$, we obtain

19 $$\begin{aligned} J_n(\varvec{\theta }) = \frac{1}{2}+\frac{1}{2n} \sum _{i=1}^n \hat{h}_{\varvec{\theta }}^{k(i)}(\mathbf {y}_i)-\frac{1}{2n} \sum _{i=1}^n \hat{h}_{\varvec{\theta }}^{k(i)}(\mathbf {x}_i). \end{aligned}$$

The function *k*(*i*) in the equation indicates to which validation set feature *i* belonged. If the Bayes classification rule is used instead of the learned $\hat{h}_{\varvec{\theta }}^{k(i)}$, we obtain $J_n^{*}(\varvec{\theta })$ in Equation (3),

20 $$\begin{aligned} J_n^{*}(\varvec{\theta }) = \frac{1}{2}+\frac{1}{2n} \sum _{i=1}^n h^{*}_{\varvec{\theta }}(\mathbf {y}_i)-\frac{1}{2n} \sum _{i=1}^n h^{*}_{\varvec{\theta }}(\mathbf {x}_i). \end{aligned}$$

The function *k*(*i*) disappeared because of the weak stationarity assumption that the marginal distributions of the $\mathbf {x}_i$ and $\mathbf {y}_i$ do not depend on *i*.

In what follows, it is helpful to introduce the set $H^{*}_{\varvec{\theta }}= \{\mathbf {u}: h^{*}_{\varvec{\theta }}(\mathbf {u})=1\}$. The normalized sums in (20) are then the fractions of features which belong to $H^{*}_{\varvec{\theta }}$. Taking the expectation over $\mathbf {X}$ and $\mathbf {Y}_{\varvec{\theta }}$, using that the expectation over the binary function $h^{*}_{\varvec{\theta }}$ equals the probability of the set $H^{*}_{\varvec{\theta }}$,

21 $$\begin{aligned} {{\mathrm{E}}}\left( h^{*}_{\varvec{\theta }}(\mathbf {y}_i)\right)&={\text {P}}_{\varvec{\theta }}\left( H^{*}_{\varvec{\theta }}\right) , \quad {{\mathrm{E}}}\left( h^{*}_{\varvec{\theta }}(\mathbf {x}_i)\right) = {\text {P}}_{\varvec{\theta }^{\circ }}\left( H^{*}_{\varvec{\theta }}\right) , \end{aligned}$$

we obtain the average discriminability ${{\mathrm{E}}}(J_n^{*}(\varvec{\theta }))=J(\varvec{\theta })$,

22 $$\begin{aligned} J(\varvec{\theta }) = \frac{1}{2} + \frac{1}{2}\left( {\text {P}}_{\varvec{\theta }}\left( H^{*}_{\varvec{\theta }}\right) - {\text {P}}_{\varvec{\theta }^{\circ }}\left( H^{*}_{\varvec{\theta }}\right) \right) . \end{aligned}$$

The difference between $J_n$ and *J* is twofold: First, relative frequencies instead of probabilities (expectations) occur. Second, learned classification rules instead of the Bayes classification rule are used.

#### Remark

There is an interesting analogy between the objective $J_n^{*}$ and the log-likelihood: The sum over the $\mathbf {y}_i$ does not depend on the observed data but on $\varvec{\theta }$ and may be considered an analogue to the log-partition function (or an estimate of it). In the same analogy, the sum over the $\mathbf {x}_i$ corresponds to the logarithm of the unnormalized model of the data. The two terms have opposite signs and balance each other as in the methods for unnormalized models reviewed by Gutmann and Hyvärinen (2013a).

### Minimization of *J*

We note that $J(\varvec{\theta }^{\circ })= 1/2$. Since $H^{*}_{\varvec{\theta }}$ contains only the points which are more probable under ${\text {P}}_{\varvec{\theta }}$ than under ${\text {P}}_{\varvec{\theta }^{\circ }}$, we have further that $J(\varvec{\theta }) \ge 1/2$. Hence, $\varvec{\theta }^{\circ }$ is a minimizer of *J*. However, $\varvec{\theta }^{\circ }$ might not be the only one: Depending on the parametrization, it could be that ${\text {P}}_{\varvec{\theta }^{\circ }}={\text {P}}_{\varvec{\theta }}$ for some $\varvec{\tilde{\theta }}$ other than $\varvec{\theta }^{\circ }$. We therefore made the identifiability assumption that the $\varvec{\tilde{\theta }}$ are well separated from $\varvec{\theta }^{\circ }$ so that there is a compact subset ${\varTheta }$ of the parameter space which contains $\varvec{\theta }^{\circ }$ but none of the $\varvec{\tilde{\theta }}$. The above can then be summarized as Proposition 2.

#### Proposition 2

$J(\varvec{\theta }^{\circ }) = 1/2$ and $J(\varvec{\theta }) > 1/2$ for all other $\varvec{\theta }\in {\varTheta }$.

Restricting the parameter space to ${\varTheta }$, consistency of $\varvec{\hat{\theta }}_n$ follows from uniform convergence of $J_n$ to *J* on ${\varTheta }$ (van der Vaart 1998, Theorem 5.7).

### Uniform convergence of $J_n$ to *J*

We show that $J_n$ converges uniformly to *J* if $J^{*}_n$ converges to *J* and if $J_n$ stays close to $J_n^{*}$ for large *n*. This splits the convergence problem into two subproblems with clear meanings which are discussed in the main text.

#### Proposition 3

23 $$\begin{aligned}&\text {If} \quad \sup _{\varvec{\theta }\in {\varTheta }} \left| J(\varvec{\theta })-J_n^{*}(\varvec{\theta })\right| \mathop {\rightarrow }\limits ^{P}0 \quad \text {and} \quad \sup _{\varvec{\theta }\in {\varTheta }} \left| J_n^{*}(\varvec{\theta })-J_n(\varvec{\theta })\right| \mathop {\rightarrow }\limits ^{P}0 \nonumber \\&\text {then}\quad \sup _{\varvec{\theta }\in {\varTheta }} \left| J(\varvec{\theta })-J_n(\varvec{\theta })\right| \mathop {\rightarrow }\limits ^{P}0. \end{aligned}$$

#### Proof

By the triangle inequality, we have

24 $$\begin{aligned} \left| J(\varvec{\theta })-J_n(\varvec{\theta })\right| \le \left| J(\varvec{\theta })-J_n^{*}(\varvec{\theta })\right| + \left| J_n^{*}(\varvec{\theta })-J_n(\varvec{\theta })\right| , \end{aligned}$$

so that

$$\begin{aligned}&\sup _{\varvec{\theta }\in {\varTheta }} \left| J(\varvec{\theta })-J_n(\varvec{\theta })\right| \nonumber \\&\quad \le \sup _{\varvec{\theta }\in {\varTheta }} \left| J(\varvec{\theta })-J_n^{*}(\varvec{\theta })\right| + \sup _{\varvec{\theta }\in {\varTheta }} \left| J_n^{*}(\varvec{\theta })-J_n(\varvec{\theta })\right| , \end{aligned}$$

and hence

25 $$\begin{aligned}&{\text {P}}\left( \sup _{\varvec{\theta }\in {\varTheta }} \left| J(\varvec{\theta })-J_n(\varvec{\theta })\right|> \epsilon \right) \nonumber \\&\quad \le {\text {P}}\left( \sup _{\varvec{\theta }\in {\varTheta }} \left| J(\varvec{\theta })-J_n^{*}(\varvec{\theta })\right| + \sup _{\varvec{\theta }\in {\varTheta }} \left| J_n^{*}(\varvec{\theta })-J_n(\varvec{\theta })\right| >\epsilon \right) \end{aligned}$$

It further holds that

26 $$\begin{aligned}&{\text {P}}\left( \sup _{\varvec{\theta }\in {\varTheta }} \left| J(\varvec{\theta })-J_n^{*}(\varvec{\theta })\right| + \sup _{\varvec{\theta }\in {\varTheta }} \left| J_n^{*}(\varvec{\theta })-J_n(\varvec{\theta })\right|>\epsilon \right) \nonumber \\&\quad \le {\text {P}}\left( \sup _{\varvec{\theta }\in {\varTheta }} \left| J(\varvec{\theta })-J_n^{*}(\varvec{\theta })\right|>\frac{\epsilon }{2}\right) \nonumber \\&\quad + {\text {P}}\left( \sup _{\varvec{\theta }\in {\varTheta }} \left| J_n^{*}(\varvec{\theta })-J_n(\varvec{\theta })\right| >\frac{\epsilon }{2} \right) \end{aligned}$$

which concludes the proof. $\square$

## Footnotes

1 Early versions were communicated as (Gutmann et al. 2014a, b).
