# Supplementary material for: Likelihood-free inference via classification
Source: Stat Comput. 2017 Mar 13;28(2):411–25. doi: 10.1007/s11222-017-9738-6 (PMC6956883; doi:10.1007/s11222-017-9738-6)
Supplement: Supplementary file 1 — Supplementary material 1 (pdf 3937 KB) [file 11222_2017_9738_MOESM1_ESM.pdf]

Likelihood-free inference via classification
# – Supplementary material –

Michael U. Gutmann MICHAEL.GUTMANN@ED.AC.UK
School of Informatics,
University of Edinburgh

Ritabrata Dutta RITABRATA.DUTTA@USI.CH
InterDisciplinary Institute of Data Science,
Universitá della Svizzera italiana

Samuel Kaski SAMUEL.KASKI@AALTO.FI
Helsinki Institute for Information Technology,
Department of Computer Science, Aalto University

Jukka Corander JUKKA.CORANDERR@MEDISIN.UIO.NO
Department of Biostatistics, University of Oslo
Helsinki Institute for Information Technology,
Department of Mathematics and Statistics, University of Helsinki

Contents

1 Models and algorithms **3**
1.1 Classification methods 3
1.2 Models used for continuous, binary, count, and time series data 3
1.3 ABC algorithm 7

2 Measuring discrepancy via classification **9**

3 Classical inference via classification **11**

4 Bayesian inference via classification **12**
4.1 The inferred posterior distributions for all classification methods used . . . 12
4.2 Movies showing the evolution of the inferred posteriors 16
4.3 Relative errors in posterior means and standard deviations 16

5 Application on real data **18**
5.1 Evolution of inferred posterior distributions on simulated data 18
5.2 Evolution of inferred posterior distributions on real data 18
5.3 Further results on compensating missing expert statistics with classifier ABC 18

List of Figures

1	Hybrid approach to choose the thresholds in classifier ABC	8
2	Measuring discrepancy via classification, Gauss (mean and variance)	9
3	Measuring discrepancy via classification, ARCH	10
4	Empirical evidence for consistency	11
5	Classifier ABC, Bernoulli	12
6	Classifier ABC, Poisson	13
7	Classifier ABC, Gauss (mean)	13
8	Classifier ABC, Gauss (mean and variance)	14
9	Classifier ABC, moving average model	15
10	Classifier ABC, ARCH model	15
11	Quantitative analysis of the inferred posterior distributions	17
12	Epidemic model, evolution of the posterior pdfs for simulated data	20
13	Zoom for the fifth generation	21
14	Epidemic model, evolution of the posterior pdfs for real data	22
15	Zoom for the fifth generation	23
16	Using classifier ABC to compensate for insufficient expert statistics	23

List of Tables

1	Links to movies showing the inference process of classifier ABC	16

1. Models and algorithms

This section contains details on the classification methods, the models for continuous, binary, count and time-series data used to test our approach, as well as the ABC algorithm employed.

1.1 Classification methods

There are many possible classification methods, ranging from traditional logistic regression to more recent deep learning and kernel methods. For an introduction, we refer the reader to the textbooks by Wasserman (2004) and Hastie et al (2009). We used methods provided by two libraries: For linear and quadratic discriminant analysis (LDA and QDA), matlab's `classify.m` was employed. For L_1 and L_2 regularized polynomial logistic regression and support vector machine (SVM) classification, we used the `liblinear` classification library (Fan et al 2008), version 1.93, via the matlab interface, with a fixed regularization penalty (we used the default value $C = 1$). The `liblinear` library is for linear classification. Polynomial classification was implemented via polynomial basis expansion (Hastie et al 2009, Chapter 5). We rescaled the covariates to the interval $[-1, 1]$ and used the first nine Chebyshev polynomials of the first kind.

For all methods but LDA, multidimensional $\mathbf{x}_i$ were projected onto their principal components prior to classification and thereafter rescaled to variance one. This operation amounts to multiplying the $\mathbf{x}_i$ with a whitening matrix, and the $\mathbf{y}_i$ were multiplied with the same matrix.

The max-rule consisted in trying several classification methods and selecting the one giving the largest classification accuracy. We used L_1 and L_2 regularized polynomial logistic regression and SVM classification with the penalties $C = 0.1, 1, 10$, as well as LDA and QDA. When LDA was not applicable (as for the moving average model), it was excluded from the pool of classification methods used for the max-rule.

1.2 Models used for continuous, binary, count, and time series data

We tested the proposed inference method on several well-known distributions. This section details the models and lists the parameters used to generate the data, as well as the priors employed for Bayesian inference and the corresponding posterior distributions. The posterior distributions served as reference against which we compared the distributions produced by classifier ABC.

The sample average of n data points $(x_1, \ldots, x_n)$ will be denoted by $\bar{x}$, and the sample variance by s_n^2,

$$\bar{x} = \frac{1}{n}\sum_{i=1}^{n} x_i, \qquad s_n^2 = \frac{1}{n}\sum_{i=1}^{n}(x_i - \bar{x})^2. \tag{S1}$$

1.2.1 Continuous data

We considered inference for a univariate Gaussian with unknown mean and known variance, and inference of both mean and variance.

Gaussian with unknown mean. The data were sampled from a univariate Gaussian with mean $\mu^o = 1$ and variance $v^o = 1$. Inference was performed on the mean μ. In the Bayesian setting, the prior distribution of μ was Gaussian,

$$\mu \sim \mathcal{N}(\mu_0, v_0), \qquad p(\mu|\mu_0, v_0) = \frac{1}{\sqrt{2\pi v_0}} \exp\left(-\frac{(\mu-\mu_0)^2}{2v_0}\right), \tag{S2}$$

with mean $\mu_0 = 3$ and variance $v_0 = 1$. For Gaussian data with known variance v^o and a Gaussian prior on the mean μ, the posterior distribution of μ is Gaussian with mean μ_n and variance v_n,

$$\mu|\mathbf{X} \sim \mathcal{N}(\mu_n, v_n), \qquad \mu_n = \left(\frac{\mu_0}{v_0} + \frac{n\bar{x}}{v^o}\right) v_n, \qquad v_n = \left(\frac{1}{v_0} + \frac{n}{v^o}\right)^{-1}, \tag{S3}$$

see, for example, (Gelman et al 2003, Chapter 2).

Gaussian with unknown mean and variance. The Gaussian data were generated with mean $\mu^o = 3$ and variance $v^o = 4$. Both mean μ and variance v were considered unknown. In the Bayesian setting, the prior distribution was normal-inverse-gamma,

$$\mu|v \sim \mathcal{N}\left(\mu_0, \frac{v}{\lambda_0}\right), \quad v \sim \mathcal{G}^{-1}(\alpha_0, \beta_0), \quad p(v|\alpha_0, \beta_0) = \frac{\beta_0^{\alpha_0}}{\Gamma(\alpha_0)} v^{-\alpha_0 - 1} \exp\left(-\frac{\beta_0}{v}\right), \tag{S4}$$

where α_0 and β_0 are the shape and scale parameters, respectively, and $\Gamma(.)$ is the gamma function, $\Gamma(t) = \int_0^\infty u^{t-1} \exp(-u) \mathrm{d}u$. The parameter values $\mu_0 = 0, \lambda_0 = 1, \alpha_0 = 3, \beta_0 = 0.5$ were used. This gives a prior variance with mean and standard deviation 0.25. The posterior is normal-inverse-gamma with updated parameters $\mu_n, \lambda_n, \alpha_n, \beta_n$,

$$\mu|v, \mathbf{X} \sim \mathcal{N}\left(\mu_n, \frac{v}{\lambda_n}\right), \quad \mu_n = \frac{\lambda_0 \mu_0 + n\bar{x}}{\lambda_0 + n}, \quad \lambda_n = \lambda_0 + n, \tag{S5}$$

$$v|\mathbf{X} \sim \mathcal{G}^{-1}(\alpha_n, \beta_n), \quad \alpha_n = \alpha_0 + \frac{n}{2}, \quad \beta_n = \beta_0 + \frac{n}{2} s_n^2 + \frac{n}{2} \frac{\lambda_0}{\lambda_0 + n} (\bar{x} - \mu_0)^2, \tag{S6}$$

see, for example, (Gelman et al 2003, Chapter 3).

1.2.2 Binary data

The data were a random sample from a Bernoulli distribution with success probability (mean) $\mu^o = 0.2$. The prior on the mean μ was a beta distribution with parameters $\alpha_0 = \beta_0 = 2$,

$$\mu \sim \mathrm{Beta}(\alpha_0, \beta_0), \qquad p(\mu|\alpha_0, \beta_0) = \frac{\Gamma(\alpha_0 + \beta_0)}{\Gamma(\alpha_0)\Gamma(\beta_0)} \mu^{\alpha_0 - 1} (1-\mu)^{\beta_0 - 1}, \tag{S7}$$

which has mean 0.5 and standard deviation 0.22. The posterior is beta with parameters α_n, β_n,

$$\mu|\mathbf{X} \sim \mathrm{Beta}(\alpha_n, \beta_n), \qquad \alpha_n = \alpha_0 + n\bar{x}, \qquad \beta_n = \beta_0 + n(1 - \bar{x}), \tag{S8}$$

see, for example, (Gelman et al 2003, Chapter 2).

1.2.3 Count data

The data were a random sample from a Poisson distribution with mean $\lambda^o = 10$. The prior on the mean λ was a gamma distribution with shape parameter $\alpha_0 = 3$ and rate parameter $\beta_0 = 1/2$,

$$\lambda \sim \mathcal{G}(\alpha_0, \beta_0), \qquad p(\lambda|\alpha_0, \beta_0) = \frac{\beta_0^{\alpha_0}}{\Gamma(\alpha_0)} \lambda^{\alpha_0 - 1} \exp\left(-\beta_0 \lambda\right). \tag{S9}$$

The prior distribution has mean 6, mode 4, and standard deviation 3.46. The posterior distribution is gamma with parameters α_n, β_n,

$$\lambda|\mathbf{X} \sim \mathcal{G}(\alpha_n, \beta_n), \qquad \alpha_n = \alpha_0 + n\bar{x}, \qquad \beta_n = \beta_0 + n, \tag{S10}$$

see, for example, (Gelman et al 2003, Chapter 2).

1.2.4 Time series

We considered a moving average and an ARCH(1) model.

Moving average model. The time series is determined by the update equation

$$x_t = \epsilon_t + \theta \epsilon_{t-1}, \quad t = 1, \ldots, T, \tag{S11}$$

where the $\epsilon_t, t = 0, \ldots, T$ are independent standard normal random variables, and ϵ_0 is unobserved. The observed data were generated with $\theta^o = 0.3$. The $\mathbf{x}_i$ for classification consisted of 2 consecutive time points (x_t, x_{t+1}).

For the derivation of the posterior distribution, it is helpful to write the update equation in matrix form. Let $\mathbf{x}_{0:T} = (x_0, \ldots, x_T)$ and $\boldsymbol{\epsilon} = (\epsilon_0, \ldots, \epsilon_T)$ be two column vectors of length $T+1$. The update equation does not specify the value of x_0. We thus set $x_0 = \epsilon_0$. Equation (S11) can then be written as

$$\mathbf{x}_{0:T} = \mathbf{B}\boldsymbol{\epsilon}, \qquad \mathbf{B} = \begin{pmatrix} 1 & 0 & & & \\ \theta & 1 & 0 & & \\ & \ddots & \ddots & \ddots & \\ & & \ddots & \ddots & 0 \\ & & & \theta & 1 \end{pmatrix}. \tag{S12}$$

It follows that $\mathbf{x}_{0:T}$ is zero mean Gaussian with covariance matrix $\mathbf{B}\mathbf{B}^\top$. Since $\mathbf{x}_{0:T}$ has a Gaussian distribution, we can analytically integrate out the unobserved x_0. The resulting vector $\mathbf{x}_{1:T}$ is zero mean Gaussian with tridiagonal covariance matrix $\mathbf{C}$,

$$\mathbf{C} = \begin{pmatrix} 1+\theta^2 & \theta & & & \\ \theta & 1+\theta^2 & \theta & & \\ & \ddots & \ddots & \ddots & \\ & & \ddots & \ddots & \theta \\ & & & \theta & 1+\theta^2 \end{pmatrix}. \tag{S13}$$

We denote the distribution of $\mathbf{x}_{1:T}$ by $p(\mathbf{x}_{1:T}|\theta)$. A uniform prior on $(-1, 1)$ was assumed for θ. The posterior probability density function of θ given $\mathbf{x}_{1:T}$ is thus $p(\theta|\mathbf{x}_{1:T})$,

$$p(\theta|\mathbf{x}_{1:T}) = \frac{p(\mathbf{x}_{1:T}|\theta)}{\int_{-1}^{1} p(\mathbf{x}_{1:T}|\theta)\mathrm{d}\theta}, \qquad \theta \in (-1, 1). \tag{S14}$$

The normalizing denominator can be computed using numerical integration. Numerical integration can also be used to compute the posterior mean and variance. We used matlab's `integral.m`.

ARCH(1) model. The model used was

$$x_t = \theta_1 x_{t-1} + \epsilon_t, \qquad \epsilon_t = \xi_t\sqrt{0.2 + \theta_2 \epsilon_{t-1}^2}, \qquad t = 1, \ldots, T, \qquad x_0 = 0, \tag{S15}$$

where the ξ_t and ϵ_0 are independent standard normal random variables. We call θ_1 the mean process coefficient and θ_2 the variance process coefficient. The observed data consist of the x_t and we generated them with $(\theta_1^o, \theta_2^o) = (0.3, 0.7)$. The $\mathbf{x}_i$ used for classification consisted of 5 consecutive time points.

For the derivation of the posterior distribution, we introduce the column vectors $\boldsymbol{\epsilon} = (\epsilon_1, \ldots, \epsilon_T)$ and $\mathbf{x}_{1:T} = (x_1, \ldots, x_T)$ which are related by a linear transformation,

$$\boldsymbol{\epsilon} = \mathbf{Q}\mathbf{x}_{1:T}, \qquad \mathbf{Q} = \begin{pmatrix} 1 & 0 & & & \\ -\theta_1 & 1 & 0 & & \\ & \ddots & \ddots & \ddots & \\ & & \ddots & \ddots & 0 \\ & & & -\theta_1 & 1 \end{pmatrix}. \tag{S16}$$

Note that the band-diagonal matrix $\mathbf{Q}$ depends on θ_1. The determinant of $\mathbf{Q}$ is one so that

$$p_{\mathbf{x}}(\mathbf{x}_{1:T}|\theta_1, \theta_2) = p_{\epsilon}(\mathbf{Q}\mathbf{x}_{1:T}|\theta_1, \theta_2). \tag{S17}$$

The assumption on the ξ_t implies that $\epsilon_t|\epsilon_{t-1}$ is Gaussian with variance $0.2 + \theta_2\epsilon_{t-1}^2$. We thus have

$$p_{\epsilon}(\boldsymbol{\epsilon}|\theta_1, \theta_2) = p_1(\epsilon_1|\theta_1, \theta_2) \prod_{t=2}^{T} \frac{1}{\sqrt{2\pi(0.2 + \theta_2\epsilon_{t-1}^2)}} \exp\left(-\frac{\epsilon_t^2}{2(0.2 + \theta_2\epsilon_{t-1}^2)}\right), \tag{S18}$$

where p_1 is the pdf of ϵ_1. Since ϵ_0 is a latent variable following a standard normal distribution, p_1 is defined via an integral,

$$p_1(\epsilon_1|\theta_1, \theta_2) = \int \frac{1}{\sqrt{2\pi(0.2 + \theta_2\epsilon_0^2)}} \exp\left(-\frac{\epsilon_1^2}{2(0.2 + \theta_2\epsilon_0^2)}\right) \frac{1}{\sqrt{2\pi}} \exp\left(-\frac{\epsilon_0^2}{2}\right) \mathrm{d}\epsilon_0. \tag{S19}$$

We used numerical integration, matlab's `integral.m`, to evaluate it. The prior distribution of (θ_1, θ_2) was the uniform distribution on the rectangle $(-1, 1) \times (0, 1)$. The posterior pdf $p(\theta_1, \theta_2|\mathbf{x}_{1:T})$ is

$$p(\theta_1, \theta_2|\mathbf{x}_{1:T}) = \frac{p_{\epsilon}(\mathbf{Q}\mathbf{x}_{1:T}|\theta_1, \theta_2)}{\int_{-1}^{1}\int_{0}^{1} p_{\epsilon}(\mathbf{Q}\mathbf{x}_{1:T}|\theta_1, \theta_2)\mathrm{d}\theta_1\mathrm{d}\theta_2}, \qquad (\theta_1, \theta_2) \in (-1, 1) \times (0, 1). \tag{S20}$$

The normalizing denominator, the posterior means and variances were computed with matlab's `integral2.m`.

1.3 ABC algorithm

There are several algorithms for approximate Bayesian computation (ABC, for an overview, see, for example, Marin et al 2012). For the results in the paper, we used a population Monte Carlo sampler, also known as sequential Monte Carlo ABC algorithm, with a Gaussian kernel (Marin et al 2012, Algorithm 4), (Beaumont et al 2009; Sisson et al 2007; Toni et al 2009). In brief, the algorithm starts with samples from the prior distribution and then produces sets (generations) of weighted independent samples where the samples from any given generation are the starting point to get the samples of the next generation. The empirical pdfs, scatter plots, and sample moments reported in the paper all take the weights into account.

In some ABC implementations, the acceptance thresholds are the empirical quantiles of the discrepancies of the accepted parameters; in others, a schedule is pre-defined. The pre-defined schedule depends on the scale of the discrepancy measure which is often unknown. Using quantiles avoids this problem, but if the quantile is set too low, too few samples will be accepted which results in a slow algorithm. For J_n, the scale is known. We took advantage of this and used a hybrid approach to choose the thresholds: The threshold for a generation was the maximum of the value given by a pre-defined schedule and the value given by the 0.1 quantile of the J_n of the accepted parameters from the previous generation. With t denoting the ABC generation, the schedule was $0.75/(1 + 0.45 \log t)$, which gives a value of 0.5 at $t = 3$.

Unlike a purely quantile-based approach, the hybrid approach avoids sudden jumps to small thresholds. We can thereby obtain posteriors for intermediate thresholds. These are faster to obtain but still informative. The final posteriors from both approaches are, however, very similar, as shown in Supplementary Figure 1.

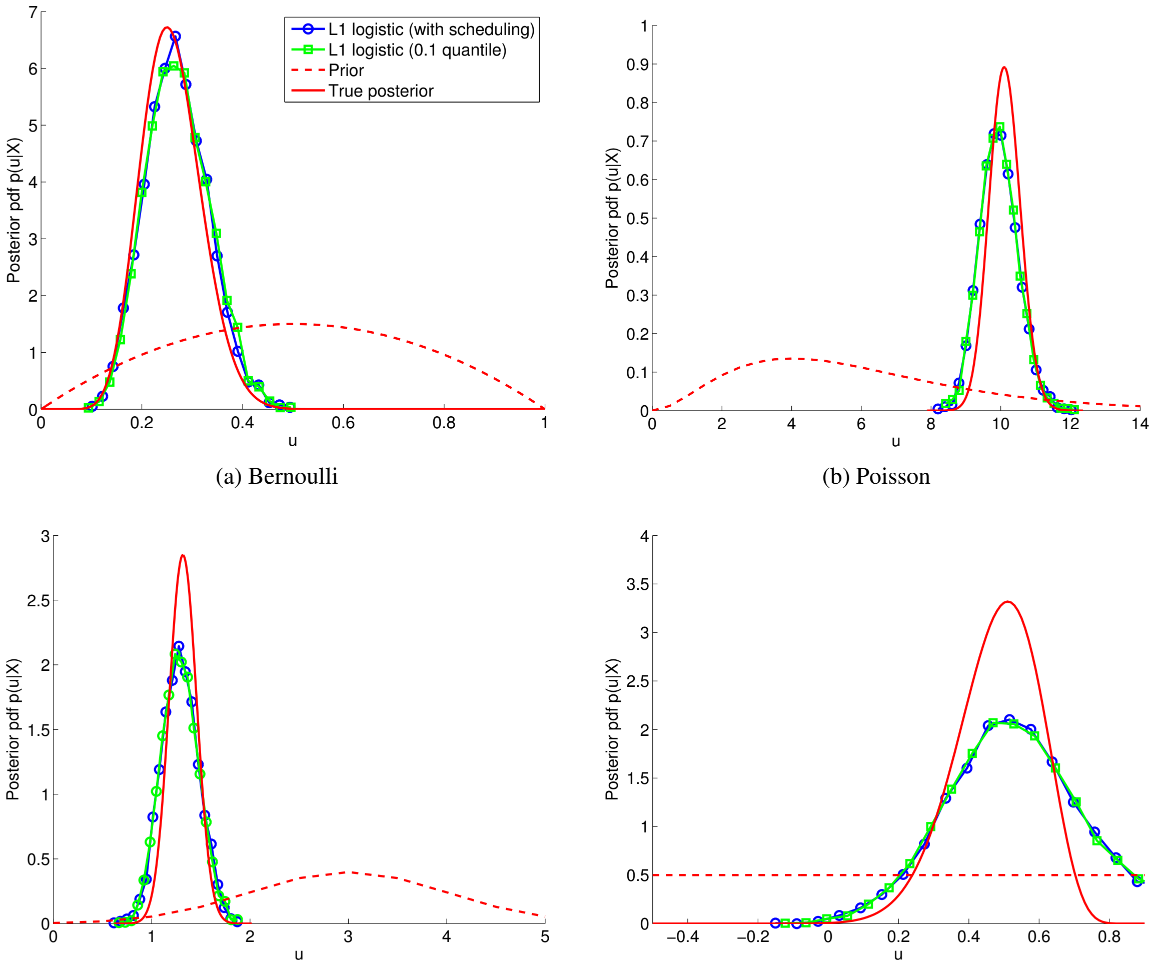

(a) Bernoulli

(b) Poisson

(c) Gauss

(d) Moving average

Supplementary Figure 1: Assessment of the hybrid approach to choose the acceptance thresholds in classifier ABC with a sequential Monte Carlo algorithm. The final posterior pdfs for the hybrid approach (blue, circles) and a purely quantile-based approach (green, squares) are very similar. The benefit of the hybrid approach is that it yields more quickly useful intermediate solutions. The results are for L_1-regularized polynomial logistic regression.

2. Measuring discrepancy via classification

In Figure 2 in the main text, chance-level discriminability was attained at a point close to the parameter $\boldsymbol{\theta}^o$ which was used to generate $\mathbf{X}$. We provide here two more such examples: Supplementary Figure 2 shows the results for a Gaussian distribution with unknown mean and variance, and Supplementary Figure 3 the results for the autoregressive conditional heteroskedasticity (ARCH) time series model in Equation (S15) with unknown mean and variance process coefficients. Parameter $\boldsymbol{\theta}^o$ is marked with a red cross.

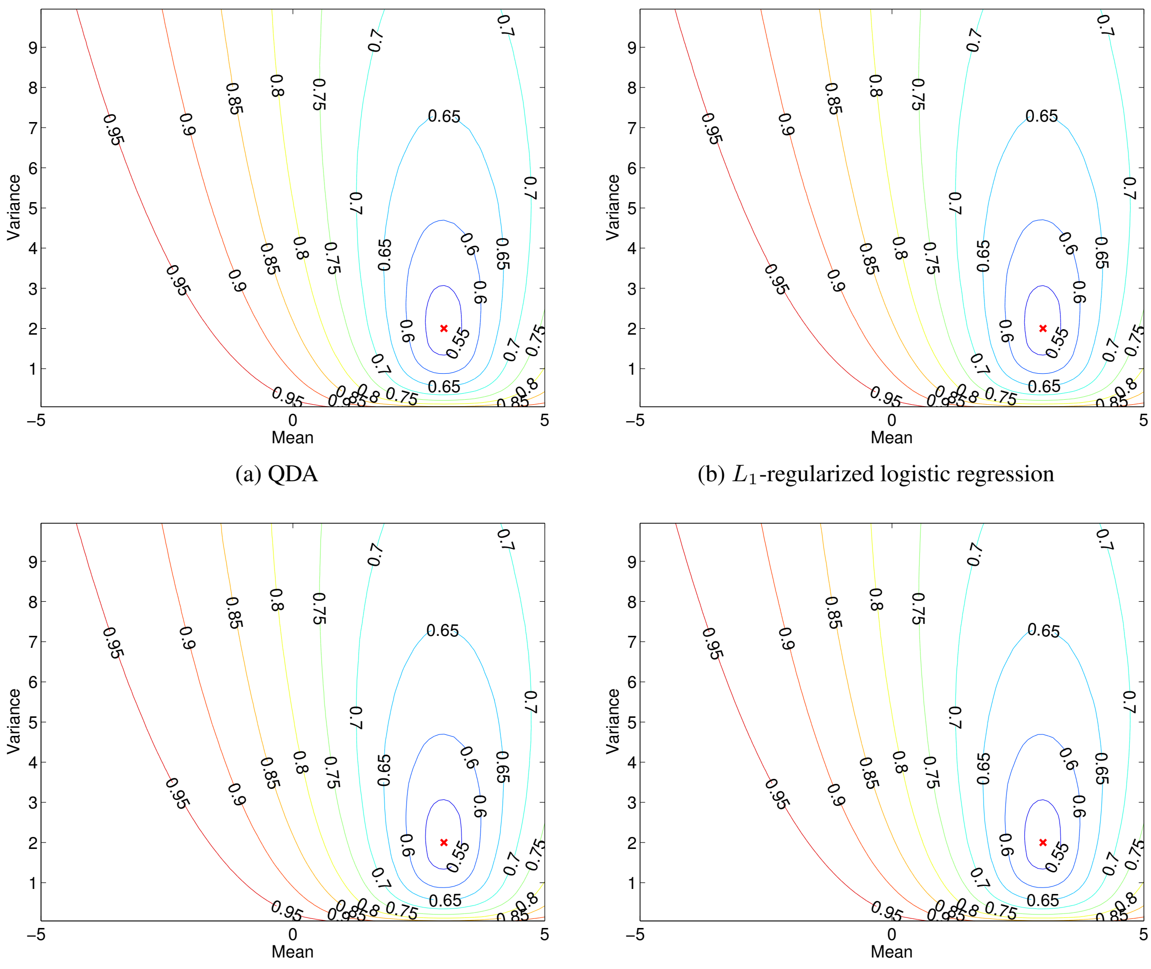

(a) QDA

(b) L_1-regularized logistic regression

(c) L_1-regularized SVM

(d) Max-Rule

Supplementary Figure 2: Gaussian with unknown mean and variance. The contour plots show J_n as a function of the two parameters for large sample sizes ($n = 100{,}000$). The different panels depict results for different classification methods. All obtain their minimal classification accuracy, chance-level discriminability 0.5, close to $\boldsymbol{\theta}^o$.

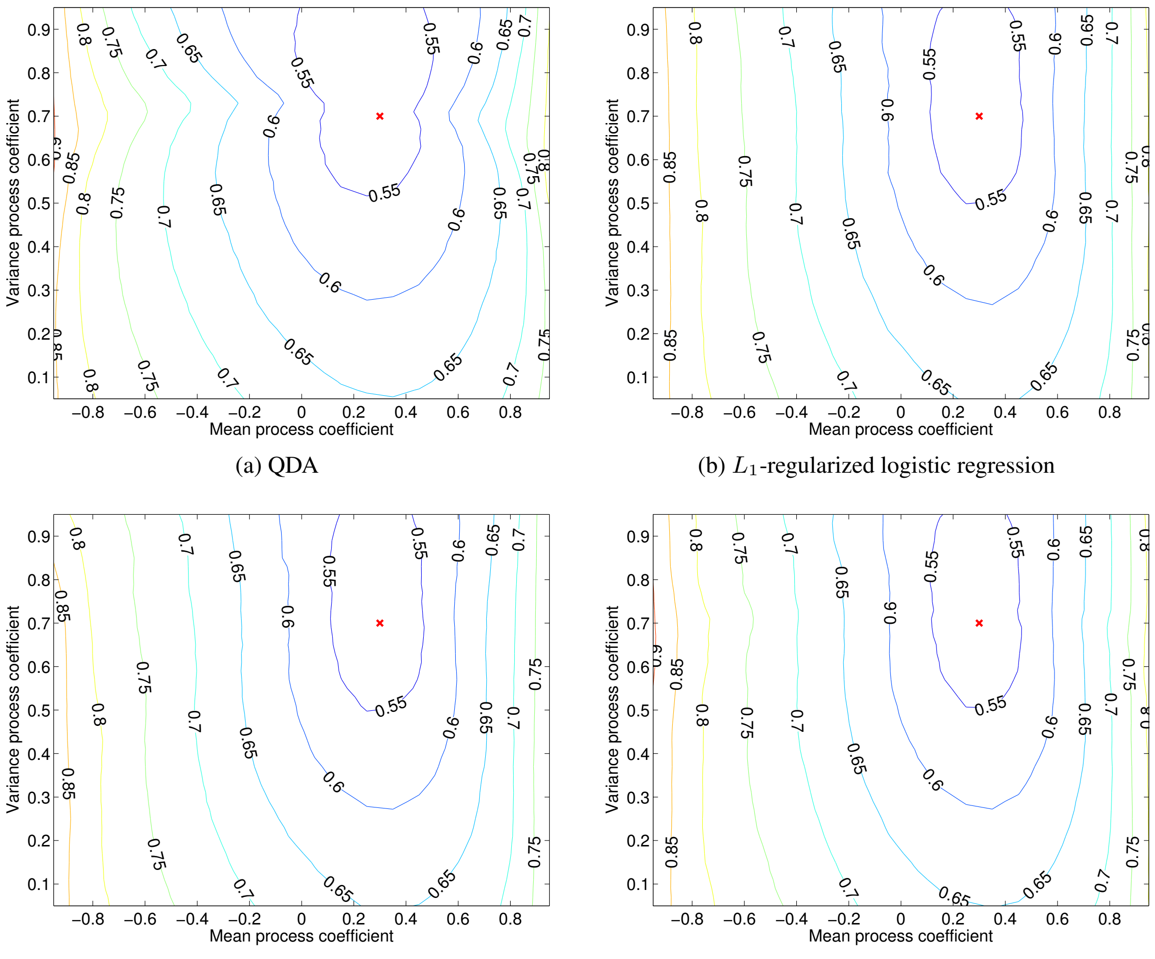

(a) QDA

(b) L_1-regularized logistic regression

(c) L_1-regularized SVM

(d) Max-Rule

Supplementary Figure 3: ARCH(1) model in Equation (S15) with unknown mean and variance process coefficients θ_1 and θ_2. The results are for $n = 10{,}000$ and visualized as in Supplementary Figure 2.

3. Classical inference via classification

In Figure 3 in the main text, we plotted the mean squared estimation error $\mathrm{E}[||\hat{\boldsymbol{\theta}}_n - \boldsymbol{\theta}^o||^2]$ for the examples in Figure 2 against the sample size n for L_1-regularized logistic regression. Supplementary Figure 4 shows the corresponding results for linear discriminant analysis (LDA), quadratic discriminant analysis (QDA), L_1-regularized polynomial support vector machine (SVM) classification, and the max-rule. As for the results in the main text, the decay is linear on the log-log scale which suggests convergence in quadratic mean, hence convergence in probability, and thus consistency of $\hat{\boldsymbol{\theta}}_n$.

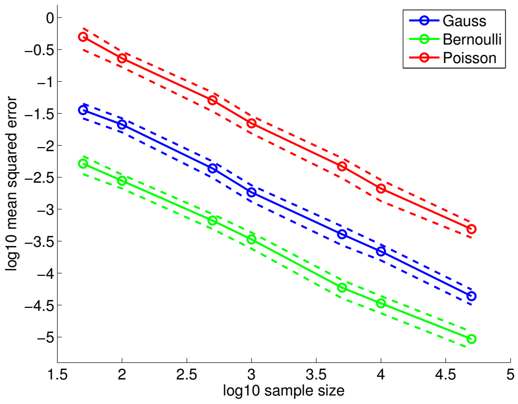

(a) LDA

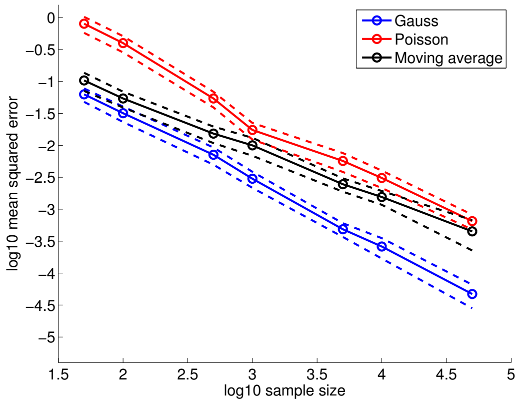

(b) QDA

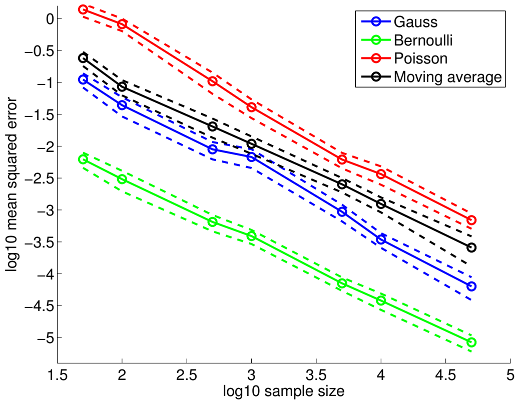

(c) L_1-regularized SVM

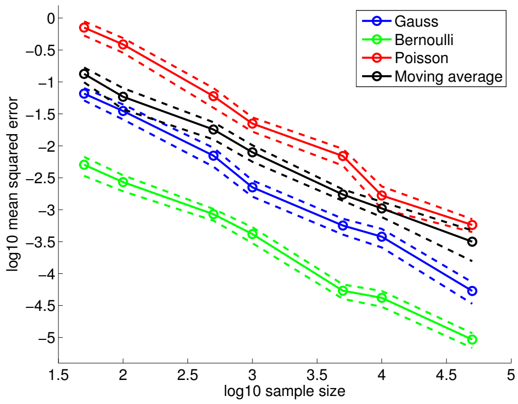

(d) Max-Rule

Supplementary Figure 4: The mean squared estimation error for the examples in Figure 2 in the main text as a function of the sample size n (solid lines, circles). The mean was computed as an average over 100 outcomes. The dashed lines depict the mean $\pm$ 2 standard errors. For QDA, the Bernoulli case is not reported because, sometimes, data with degenerate covariance matrices were generated, which the standard QDA algorithm used was not able to handle. For LDA, the moving average case was omitted since LDA cannot approximate its Bayes classification rule as discussed in the main text. The linear trend on the log-log scale suggests convergence in quadratic mean, and hence consistency of the estimator $\hat{\boldsymbol{\theta}}_n$.

4. Bayesian inference via classification

This section contains further results for classifier ABC on data with known properties, supplementing Section 4 of the main text.

4.1 The inferred posterior distributions for all classification methods used

We report the posterior distributions for all classification methods used in the paper in Supplementary Figure 5 to Supplementary Figure 10. The results are organized according to the modality of the data.

The results are for $n = 50$ and 10,000 ABC samples with a sequential Monte Carlo implementation of ABC. For the univariate cases, empirical pdfs of the ABC samples are shown together with the reference posterior pdf (red solid) and the prior pdf used (red dashed). For the bivariate cases, the ABC samples are shown as a scatter plot and the reference posterior is visualized using contour plots (red solid line). The priors are either shown as contour plots (with red dashed lines) or, if uniform, by hatching their domain.

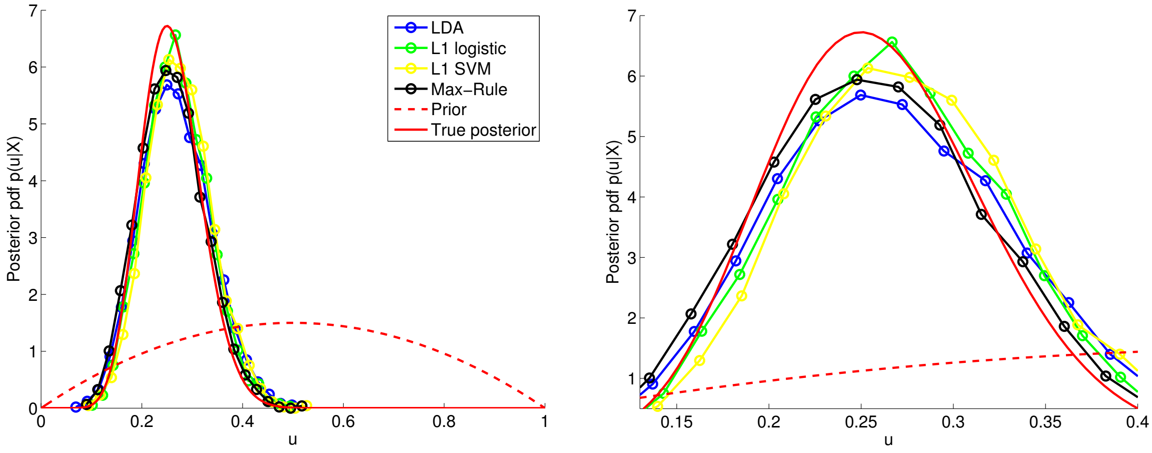

(a) Prior and posterior

(b) Zoom

Supplementary Figure 5: Binary data: Inferred posterior distribution of the success probability of a Bernoulli random variable.

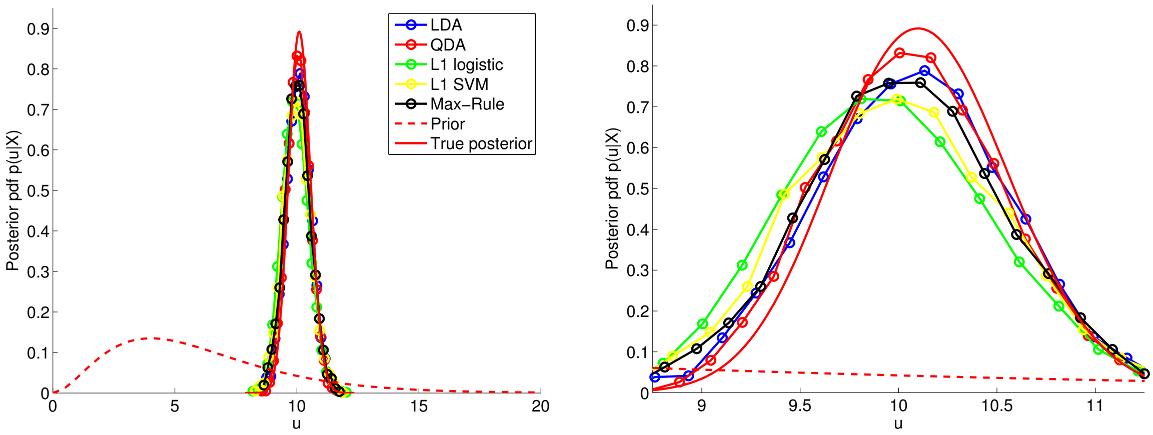

(a) Prior and posterior

(b) Zoom

Supplementary Figure 6: Count data: Inferred posterior distribution of the mean of a Poisson random variable.

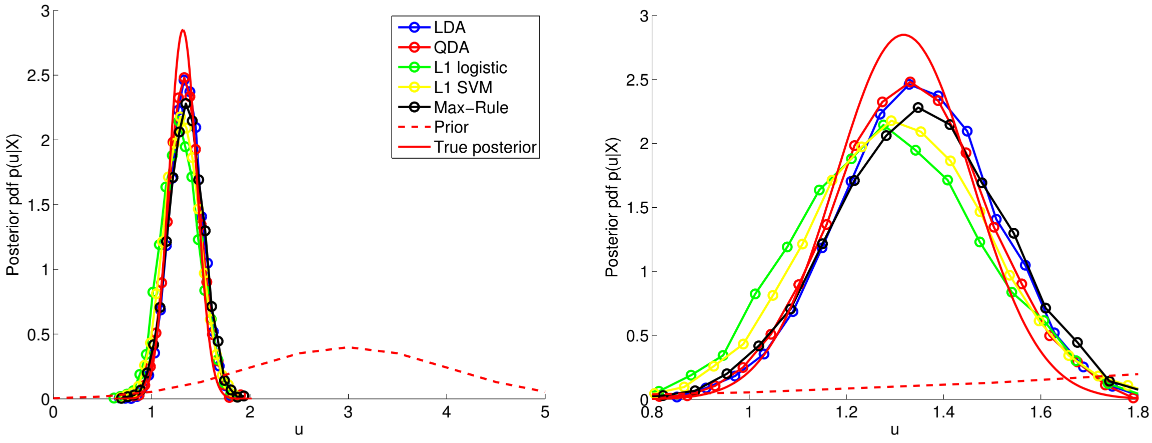

(a) Prior and posterior

(b) Zoom

Supplementary Figure 7: Continuous data: Inferred posterior distribution of the mean of a Gaussian random variable with known variance.

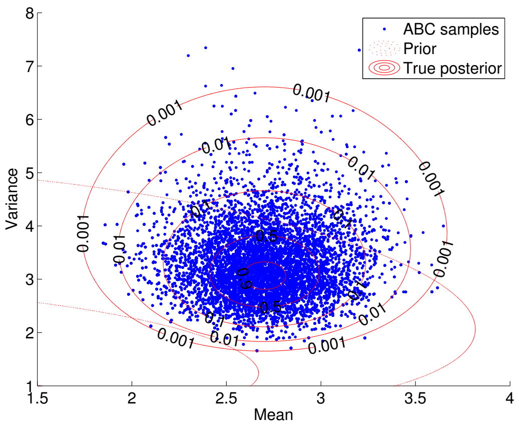

(a) QDA

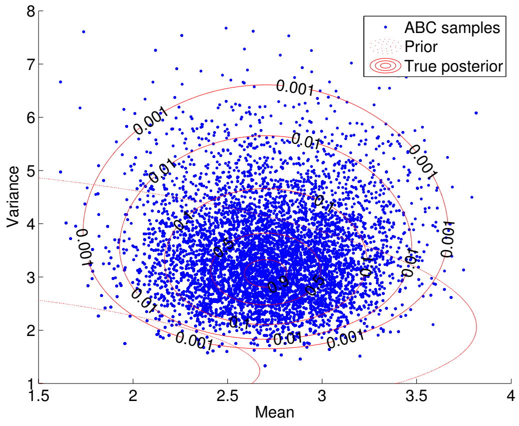

(b) L_1-regularized logistic regression

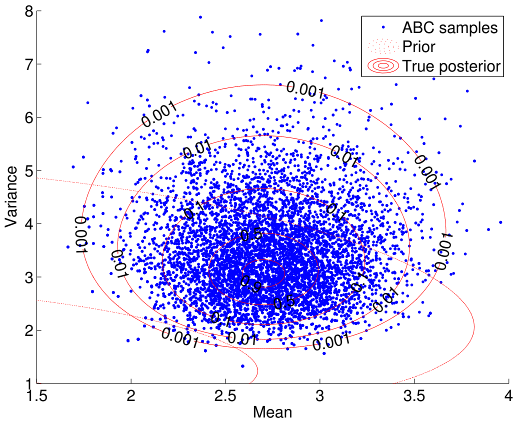

(c) L_1-regularized SVM

Supplementary Figure 8: Continuous data: Inferred posterior distribution of the mean and variance of a Gaussian random variable.

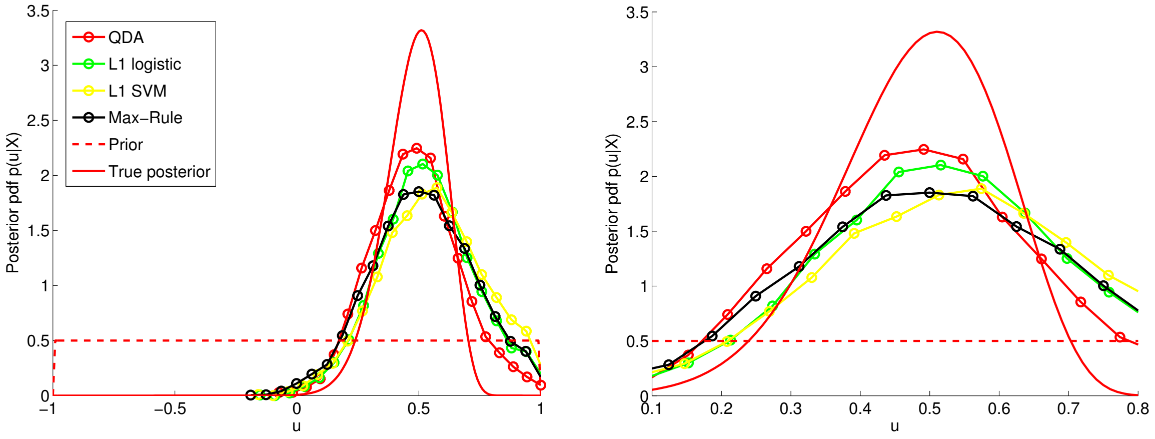

(a) Prior and posterior

(b) Zoom

Supplementary Figure 9: Time series: Inferred posterior distribution of the lag coefficient of a zero mean moving average model of order one.

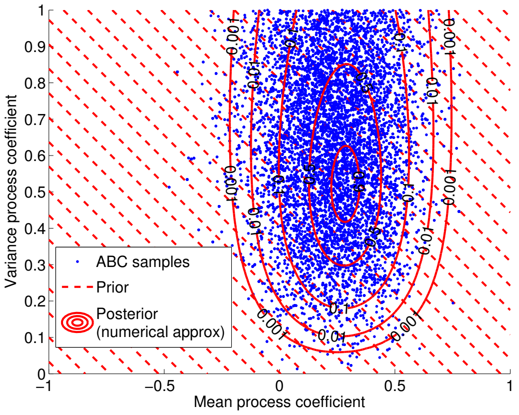

(a) QDA

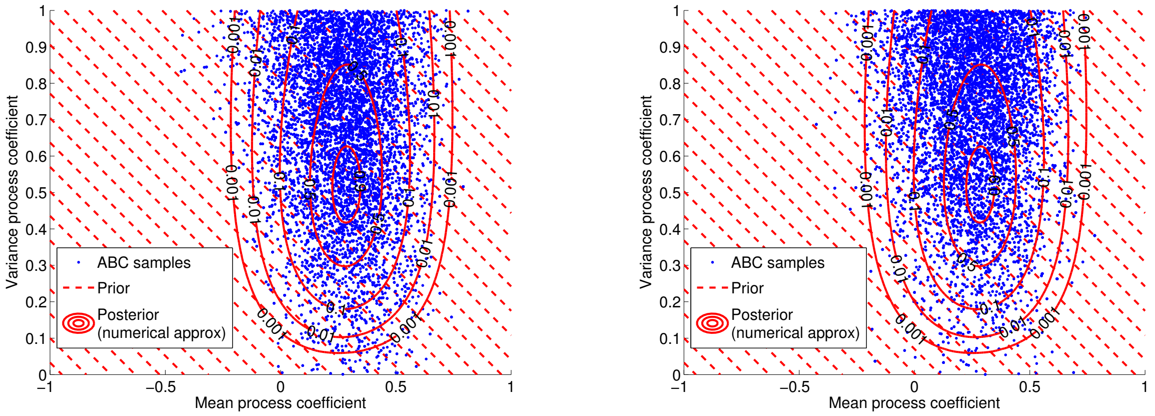

(b) L_1-regularized logistic regression

(c) L_1-regularized SVM

Supplementary Figure 10: Time series: Inferred posterior distribution of the mean and variance process coefficients of a ARCH(1) model.

4.2 Movies showing the evolution of the inferred posteriors

The sequential Monte Carlo algorithm which we used together with classifier ABC is iteratively morphing a prior distribution into a posterior distribution. Table 1 contains links to movies which show this process.

Data	LDA	QDA	Logi regr	SVM	Max-Rule
Binary (Bernoulli)	avi mp4		avi mp4	avi mp4	avi mp4
Count (Poisson)	avi mp4	avi mp4	avi mp4	avi mp4	avi mp4
Continuous (Gauss, mean)	avi mp4	avi mp4	avi mp4	avi mp4	avi mp4
Continuous (Gauss, mean & var)		avi mp4	avi mp4	avi mp4	avi mp4
Time series (moving average)		avi mp4	avi mp4	avi mp4	avi mp4
Time series (ARCH)		avi mp4	avi mp4	avi mp4	avi mp4

Table 1: Links to movies showing the inference process of classifier ABC with a sequential Monte Carlo algorithm. Online at `https://www.cs.helsinki.fi/u/gutmann/material/CLAD/movies/`

4.3 Relative errors in posterior means and standard deviations

As a quantitative analysis, we computed the relative error in the mean and the standard deviation of the inferred posterior distributions. The comparison is based on the mean and standard deviation of the true posterior if available, or, if not, the posterior obtained by deterministic numerical integration, see Supplementary material 1.2.

Supplementary Figure 11 shows the relative error for the max-rule as a function of the iteration in the ABC algorithm. The error stabilizes within 4-5 iterations. For the examples with independent data points, the errors in the posterior mean are within 5% after stabilization. A larger error of 15% occurs for the time series data. The histograms and scatter plots show, however, that the corresponding ABC samples are still very reasonable.

While the relative error for the mean is both positive or negative, for the standard deviation, the error is positive only. This means that the inferred posteriors have a larger spread than the reference posteriors, that is, the posterior variance is overestimated. Further, the relative errors are generally larger for the standard deviations than for the means. This may not be too surprising though: Also in the framework of maximum likelihood estimation, the variance of the estimate of the variance is twice the variance of the estimate of the mean for standard normal random variables.

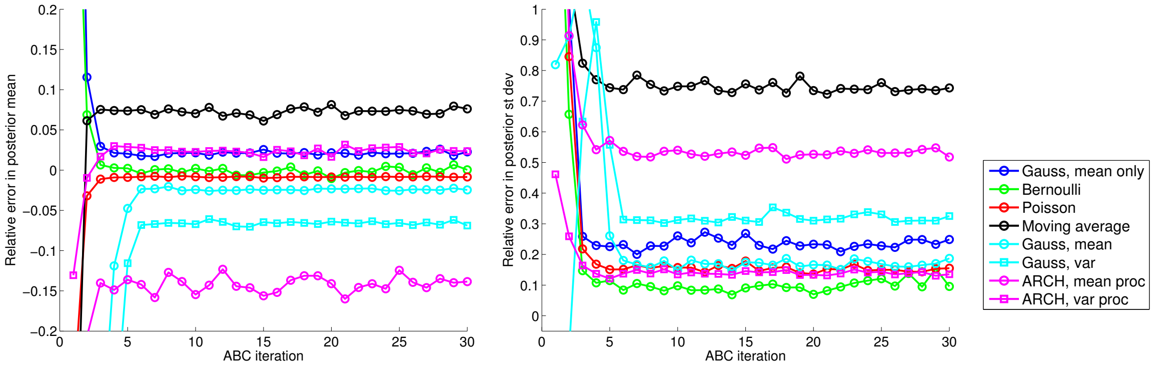

(a) Relative error in posterior mean

(b) Relative error in posterior standard deviation

Supplementary Figure 11: Quantitative analysis of the inferred posterior distributions. The curves show the relative error in the posterior mean and standard deviation for the Gauss, Bernoulli, Poisson, moving average, and ARCH examples. The results are for classification with the max-rule.

5. Application on real data

This section supplements Section 5 of the main text. Further results and analysis of our application to infectious disease epidemiology are presented.

5.1 Evolution of inferred posterior distributions on simulated data

We inferred the individual-based epidemic model with a sequential Monte Carlo ABC with J_n as discrepancy measure (classifier ABC). Supplementary Figure 12 visualizes the evolution of the inferred posterior distribution over four generations. We show the results for classifier ABC with random subsets (blue, circles) and without (red, squares). For reference, the results with the method by Numminen et al (2013), which uses expert knowledge, are shown in black (point markers). Figure 7 in the main text shows the fourth generation results in greater detail.

Numminen et al (2013) presented posterior distributions for four generations. In both the results reported here and the results by Numminen et al (2013), the mean of the inferred posteriors seems to have stabilized after four generations. The spread of the inferred posteriors, however, is still slightly shrinking. We thus ran the simulations for an additional fifth iteration. The results are shown in Supplementary Figure 13. With the fifth iteration, the posterior pdfs for classifier ABC with random projections became more concentrated and also more similar to the expert solution than the posteriors of classifier ABC without random projections. The smaller posterior variance is in line with the tighter J_n-diagrams in Figure 6 in the main text.

5.2 Evolution of inferred posterior distributions on real data

The evolution of the posterior pdfs during the ABC algorithm is shown in Supplementary Figure 14. Starting from uniform distributions, posterior distributions with well defined modes emerged. Figure 8 in the main text shows the fourth generation results in greater detail. While the posteriors of Λ and θ are qualitatively similar for all three methods, the posterior of β has a smaller mode for classifier ABC with random subsets (blue, crosses) than for classifier ABC without random subsets (red, asterisks) or the expert solution (black, plus markers). This behavior persists in the fifth generation as shown in Supplementary Figure 15. Compared to the fourth generation results, the posteriors for classifier ABC with random subsets (blue, crosses) and the expert solution (black, plus markers) became in the fifth generation more concentrated than the posterior for classifier ABC without random subsets (red, asterisks).

5.3 Further results on compensating missing expert statistics with classifier ABC

Classifier ABC, or more generally the discrepancy measure J_n, is able to incorporate expert statistics, by letting them be features (covariates) in the classification. On the one hand, this allows for expert knowledge to be used in classifier ABC. On the other hand, it allows one to enhance expert statistics by data-driven choices. The latter is particularly important if only a insufficient set of summary statistics may be specified. We show here that classifier ABC can counteract shortcomings caused by a suboptimal choice of expert statistics, thereby making the inference more robust.

We selected two (simple) expert statistics used by Numminen et al (2013), namely the number of different strains circulating and the proportion of individuals who are infected. We then inferred the posteriors with this reduced set of summary statistics only, using the method of Numminen et al (2013). Supplementary Figure 16 visualizes the resulting posterior pdfs (curves in magenta with diamond markers). A comparison with the expert solution with a full set of summary statistics (black curve, point markers) shows that the posterior distributions of Λ and θ are affected by the suboptimal choice of expert statistics. We then included the two selected expert statistics as additional features in classifier ABC. Consequently, the posteriors of Λ and θ recuperated, both when random features were present (cyan curve with triangles) or not (red curve with hexagrams).

References

Beaumont M, Cornuet JM, Marin JM, Robert C (2009) Adaptive approximate Bayesian computation. Biometrika 96(4):983–990

Fan RE, Chang KW, Hsieh CJ, Wang XR, Lin CJ (2008) Liblinear: A library for large linear classification. Journal of Machine Learning Research 9:1871–1874

Gelman A, Carlin J, Stern H, Rubin D (2003) Bayesian Data Analysis. Chapman & Hall

Hastie T, Tibshirani R, Friedman J (2009) The Elements of Statistical Learning. Springer

Marin JM, Pudlo P, Robert C, Ryder R (2012) Approximate Bayesian computational methods. Statistics and Computing 22(6):1167–1180

Numminen E, Cheng L, Gyllenberg M, Corander J (2013) Estimating the transmission dynamics of Streptococcus pneumoniae from strain prevalence data. Biometrics 69(3):748–757

Sisson S, Fan Y, Tanaka M (2007) Sequential Monte Carlo without likelihoods. Proceedings of the National Academy of Sciences 104(6):1760–1765

Toni T, Welch D, Strelkowa N, Ipsen A, Stumpf M (2009) Approximate Bayesian computation scheme for parameter inference and model selection in dynamical systems. Journal of the Royal Society Interface 6(31):187–202

Wasserman L (2004) All of statistics. Springer

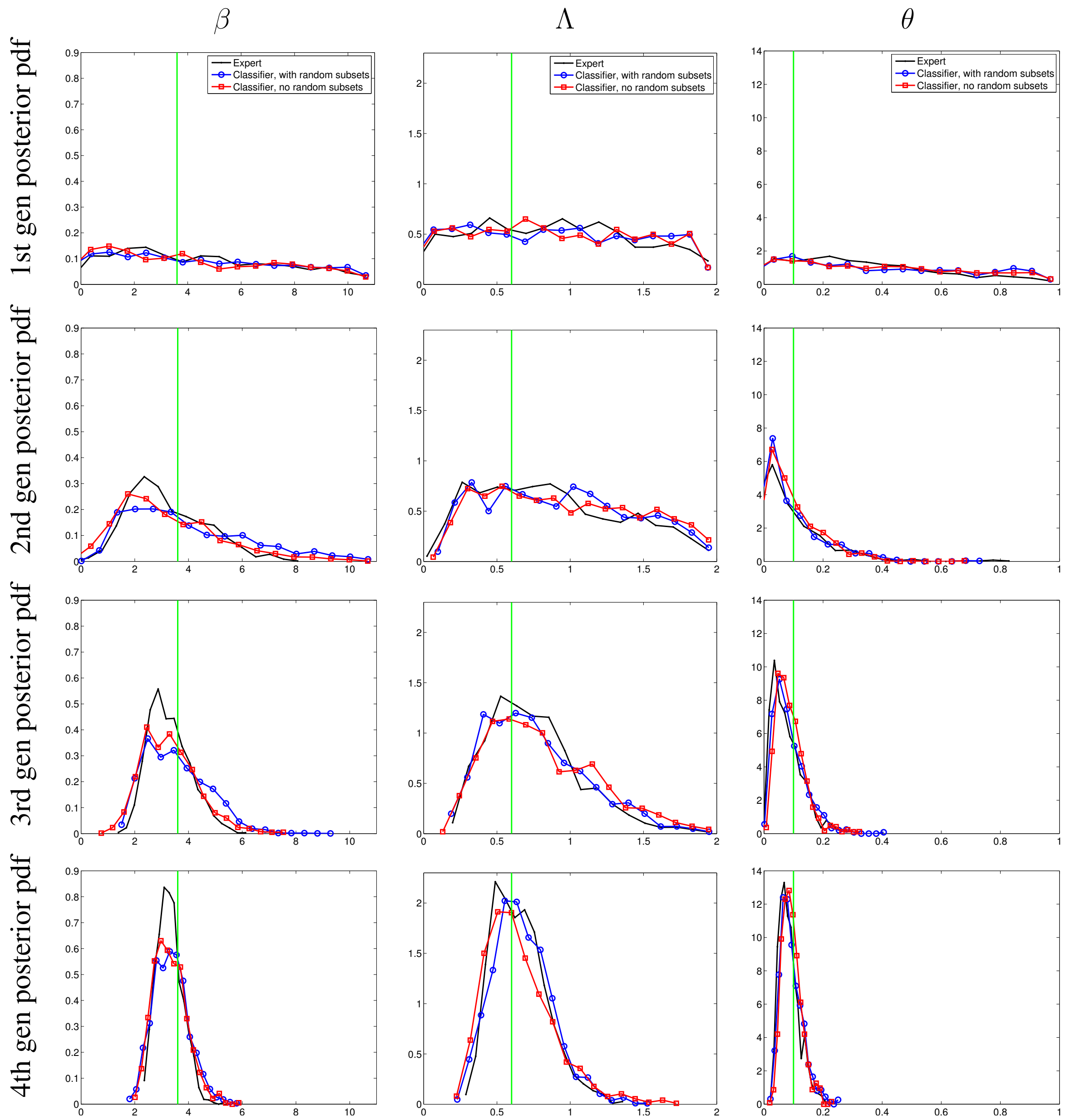

Supplementary Figure 12: Simulated data: Evolution of the posterior pdfs (scaled histograms of the samples). Black, points: ABC solution using expert knowledge, produced with code from Numminen et al (2013). Blue, circles: classifier ABC with random subsets. Red, squares: classifier ABC without random subsets. Green vertical lines: location of the data generating parameter $\boldsymbol{\theta}^o$. The results are for 1,000 ABC samples.

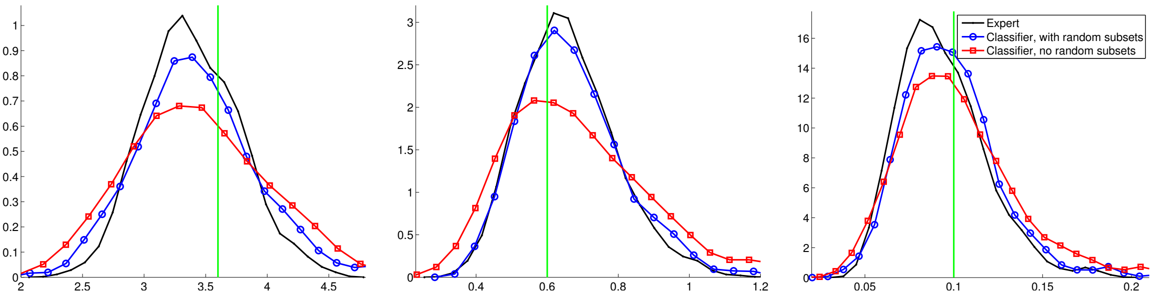

(a) Posterior pdf for β (b) Posterior pdf for Λ (c) Posterior pdf for θ

Supplementary Figure 13: Simulated data: Fifth generation results. The posterior pdfs are kernel density estimates based on 1,000 ABC samples. We used matlab's ksdensity.m with the default settings, that is, a Gaussian kernel with an adaptively chosen bandwidth. Classifier ABC with random projections (blue, circles) yielded results which are more similar to the expert solution (black, points) than classifier ABC without random projections (red, squares).

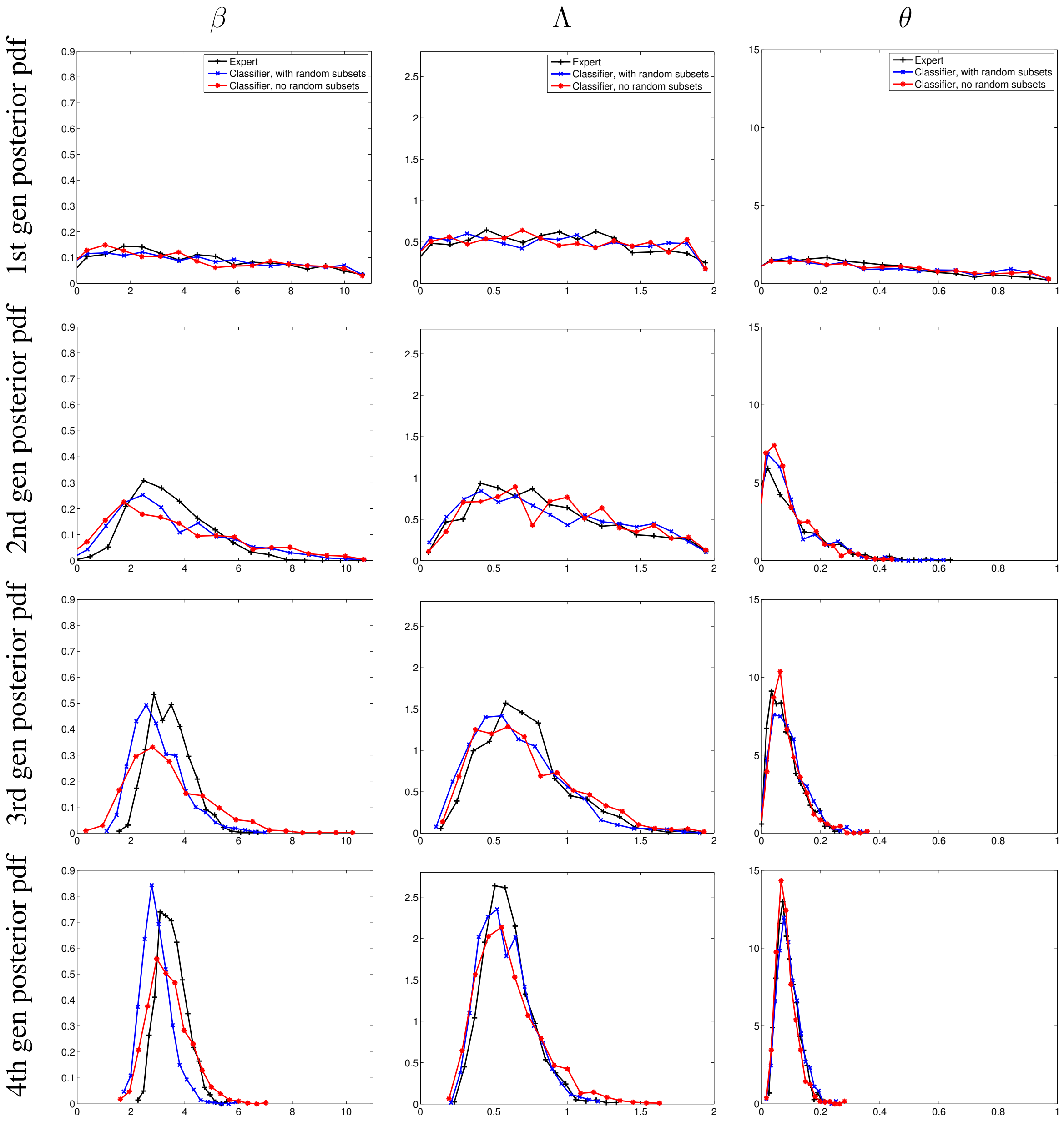

Supplementary Figure 14: Real data: Evolution of the posterior pdfs (scaled histograms of the samples). Black, plus markers: ABC solution using expert knowledge, produced with code from Numminen et al (2013). Blue, crosses: classifier ABC with random subsets. Red, asterisks: classifier ABC without random subsets. The results are for 1,000 ABC samples.

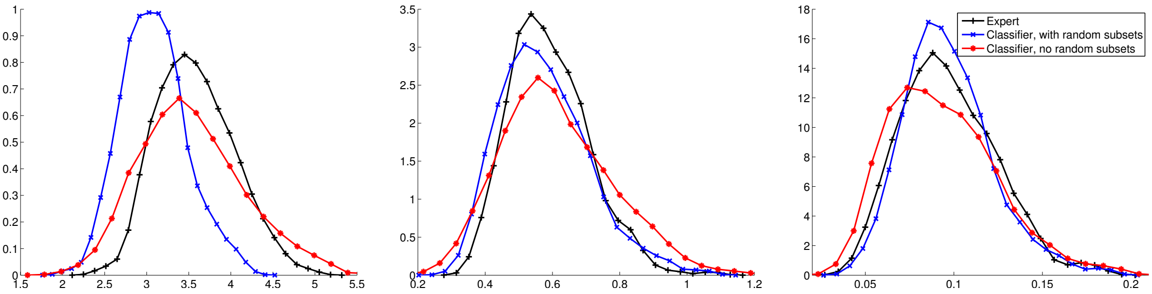

(a) Posterior pdf for β (b) Posterior pdf for Λ (c) Posterior pdf for θ

Supplementary Figure 15: Real data: Fifth generation results. The posterior pdfs are kernel density estimates based on 1,000 ABC samples. We used matlab's ksdensity.m with the default settings, that is, a Gaussian kernel with an adaptively chosen bandwidth. The posteriors for classifier ABC with random subsets (blue, crosses) and the expert solution (black, plus markers) are more concentrated than the posterior for classifier ABC without random subsets (red, asterisks).

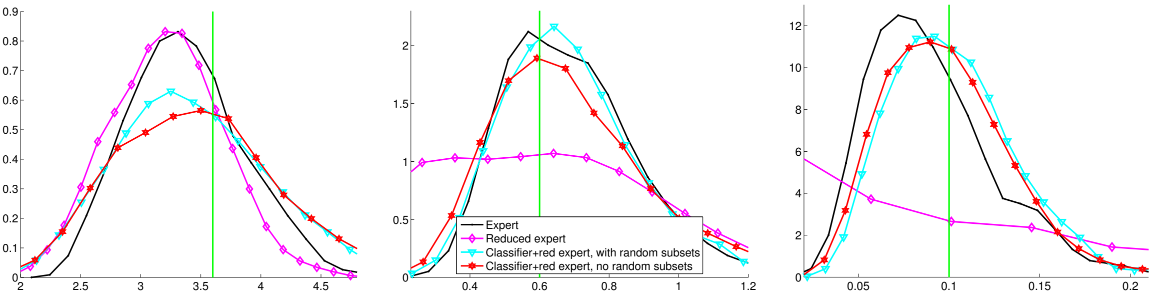

(a) Posterior pdf for β (b) Posterior pdf for Λ (c) Posterior pdf for θ

Supplementary Figure 16: Using expert statistics in classifier ABC. The results are for simulated data and show the fourth generation pdfs. Visualization is as in e.g. Supplementary Figure 15. ABC with a reduced set of expert statistics affected the posteriors (black curve with points vs magenta curve with diamonds as markers). Classifier ABC was able to counteract the shortcomings caused by the suboptimal choice of expert statistics (cyan curve with triangles and red curve with hexagrams).
